# Improved Optical Performance Analysis of YAG:Ce, NCS:Sm, and CAO:Mn Phosphors Physically Integrated with Metal–Organic Frameworks

**DOI:** 10.1021/acsomega.4c07786

**Published:** 2024-10-09

**Authors:** Sibel Oguzlar, Merve Zeyrek Ongun, Pelin Köse Yaman, Mustafa Erol

**Affiliations:** † Dokuz Eylul University, Center for Fabrication and Application of Electronic Materials, 35390 Izmir, Turkey; ‡ Dokuz Eylul University, Izmir Vocational High School, Chemistry and Chemical Processing Technologies Department, Chemical Technology Program, 35380 Izmir, Turkey; § Dokuz Eylul University, Department of Chemistry, Faculty of Science, 35390 Izmir, Turkey; ∥ Dokuz Eylul University, Department of Metallurgical and Materials Engineering, 35390 Izmir, Turkey

## Abstract

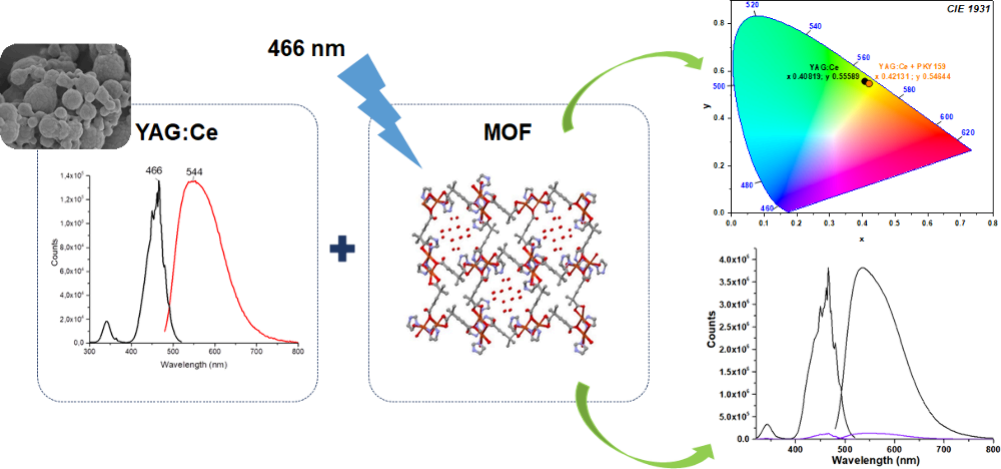

This research presents a thorough examination of the optical properties and performance enhancement strategies of synthesized phosphors, namely, yttrium aluminum garnet doped with cerium (YAG:Ce), sodium calcium silicate with samarium (NCS:Sm), and calcium aluminate oxide doped with manganese (CAO:Mn). The study delves into the synthesis processes of the phosphors, illumination of the crystal structures, and enhancement of luminescent characteristics. Additionally, the paper extends to the synthesis and analysis of {[Cu(μ_3_-dmg)(im)_2_]·3H_2_O}*_n_* (PKY159), and the coordination polymer (CP) was added the phosphors to explore a novel approach for enhanced optical performance. When the phosphor composites YAG:Ce, CAO:Mn, and NCS:Sm were made as poly(methyl methacrylate) (PMMA; for homogenization, stabilization) thin films with the coordination polymer PKY159 included, the intensity values increased by 97%, 96%, and 79%, respectively, in comparison to their pristine form. Also, all phosphors along with the PKY159 additive were examined for their decay time kinetics, thermal stabilities, CIE chromaticity coordinates, and quantum efficiencies. The YAG:Ce, NCS:Sm, and CAO:Mn mixes exhibit good thermal stability in addition to internal quantum efficiency (IQE) values that are much higher than the phosphors’ additive-free form (95.6%, 66.3%, and 84.6%, respectively). This increase is correlated to an increase in steady-state measurements. These comprehensive analyses contribute valuable insights into the design and optimization of phosphor and coordination polymer blends for improved optical functionality.

## 1 Introduction

Phosphor materials, which influence the development of new devices and circuits in the electronics, communication, and optoelectronics industries with their luminescence properties, are indispensable for various applications from lighting technologies to sensor devices.^1,2^ The increasing demand for phosphor materials encourages researchers to conduct comprehensive scientific and technological research to create phosphors with superior properties.^3−5^ By experimenting with different doping methods, including hybrid materials and alloys, researchers are investigating new material compositions for phosphors with superior optical data such as wider color ranges, higher quantum efficiency, and improved stability.^6−8^ Optimization of phosphors used in various optoelectronic applications results in increased energy efficiency as a result of the emission of desired wavelengths.^2,9,10^

By using the luminescence and color tuning properties to enhance the efficiency and visual performance of various optoelectronic devices, phosphor materials including yttrium aluminum garnet doped with cerium (yaG:Ce), sodium calcium silicate with samarium (NCS:Sm), and calcium aluminate oxide doped with manganese (CAO:Mn) play essential roles in modern lighting and display technologies.^11−13^ Because YAG:Ce has a high efficiency of converting blue light emitting diode (LED) light to yellow, which produces a white light source, it is a significant phosphor material that is widely utilized in LEDs and solid-state lighting.^14^ Song et al. reported that samples containing PVA and PVP spacers had photoluminescence (PL) development rates of 1.75 and 2.08, respectively, when they employed them as spacers between Ag NPs and the YAG:Ce phosphor substrate.^15^ Wu et al.’s study reported that the YAG:Ce intensity increased twice with SiO_2_ doping compared to the undoped one.^16^ According to Ueda et al., Yb^3+^ enhanced the superlong persistent brightness of Y_3_A_l2_Ga_3_O_12_:Ce^3+^ phosphor.^17^ Another important phosphor material frequently used in phosphor-converted LEDs and displays is NCS:Sm. Used to create efficient and visually appealing lighting solutions, NCS:Sm phosphors play a crucial role in converting the blue or UV light emitted by the LED into different colors, such as white light or various colors for displays.^18^ Red light-emitting LEDs use a luminous phosphorescent substance called CAO:Mn, which has excellent thermal stability in illumination and display technologies.^19^ According to the literature, the photoluminescence of CAO:Mn increases significantly with the addition of MgO.^20,21^ The improvement of Ca_0.96_Zn_0.04_Al_12_O_19_:0.5%Mn^4+^ emission intensity was seen up to 2.2 times that of pure CaAl_12_O_19_:0.5%Mn^4+^ when the doping concentration of Zn^2+^ was 4%.^22^

Coordination polymers (CPs) or metal–organic frameworks (MOFs) consist of organic ligands and metal ions that diffuse through coordinating bonds and other weak chemical interactions.^23−25^ CPs have gained significant attention for their exceptional functional properties, with applications spanning gas storage and separation, catalysis, optics, electronics, drug delivery, conductivity, magnetism, and more. Notably, numerous luminescent coordination polymers show potential as phosphors for LED applications. When phosphors used for LEDs interact with coordination polymers (CPs) used as effective hosts, their optical quality can be significantly improved, increasing the width of phosphor emission and resulting in a brighter and more vibrant light output.^26−28^ Moreover, the inherent protective properties of CPs play a crucial role in extending the lifetime of the phosphor and maintaining the durability of the optical properties. The interaction between CPs and phosphors shows promise in their use for applications in LEDs and displays.^29−31^ This integration accelerates the advancement of energy-efficient lighting technologies by providing improved energy efficiency, superior color rendering, and increased efficiency.^26,32^ Chen and colleagues investigated the availability of encapsulation of rhodamine in an adenine-based bio metal–organic framework (MOF) to turn various MOF composites with variable rhodamine ratios on the effects such as color tunability, emission efficiency, and emission lifetimes. The resulting composites provide possible applications in visible light communications and light-emitting devices, providing high quantum rate color tunability (up to 79%).^33^ Chen et al. synthesized a new red-emitting cationic iridium(III)-coordinated polymer and found that GaN-based LEDs using only Y_3_Al_5_O_12_:Ce^3+^ (YAG:Ce, 7.0 wt % in silicon) can whiten in the cold because this coordinating polymer can become hotter when phosphors break down. The mixture showed that GaN could be efficiently tailored with light and a blue beam.^34^ In addition, Table 1 lists studies in the literature on different phosphors and MOF materials with or without additives.

**Table 1:** Literature Search of Compositional Studies on the Phosphors

Phosphor/MOF	Additive	λ_max_^ex^	λ_max_^em^	IQE (%)/QY (%)	Obtained Result	Ref
YAG:Ce^3+^	Ni:SnO_2_	466	540	94.1%	*I*/*I*_0_ = 59%	(8)
CaAl_12_O_19_:Mn^4+^ (CAO:Mn)	Ni:SnO_2_	466	654	80.4%	*I*/*I*_0_ = 93%	
Na_2_CaSiO_4_	Sm^3+^	402	602	-	display intense emission	(13)
YAG:Ce^3+^	SiO_2_	456	527	-	2 times as high according to free YAG:Ce^3+^	(16)
YAG:Ce^3+^	cationic iridium(III) coordination polymer	457	588	-	adding a coordination polymer transformed older wLEDs into warm white light	(34)
YAG:Ce^3+^	TiO_2_	466	566	97.6%	*I*/*I*_0_ = 220%	(35)
YAG:Ce^3+^	TiO_2_:Ce^3+^	466	566	96.9%	*I*/*I*_0_ = 140%	
Y_2_O_3_:Sm^3+^	Eu^2+^	423	660	-	enhanced red emission	(36)
Y_2_O_3_:Eu^3+^	-	250	611.6	-	at 8 mol % Eu^3+^, cubic nanospheres show strong red photoluminescence	(37)
Y_2_O_3_:Eu^3+^	-	266	611; 624	-	Y_2_O_3_:Eu^3+^ nanosheets offer potential for GaAlN-based white LEDs	(38)
Na_2_CaSiO_4_	Eu^3+^	393	613	58.9%	-	(39)
CaAl_12_O_19_:Mn^4+^ (CAO:Mn)	Mg^2+^	340; 460	654	56.1%	-	(40)
[Ir(ppy)_2_(bpy)]+@1 composite	-	370	530	20.4%	tunable emission and significantly improved quantum efficiency	(41)
[Zn_4_OL_2_·*x*DMF]*n* (1) (carbazole-based blue-light-emitting MOF)	4-(dicyanomethylene)-2-methyl-6-(*p*-dimethylaminostyryl)-4*H*-pyran (DCM) and coumarin 6 (C6)	371	431	39.4%	high-performance white light emitter	(42)

The investigations mentioned earlier make it clear that the spectral characteristics of these phosphors have typically been investigated separately rather than in combination with other phosphors or metal–organic frameworks (MOFs). As such, it is worthwhile to investigate the spectrum performance of phosphors as blends. The superior optical transparency in the visible range, high light guiding, excellent resistance to sunlight exposure, and possible uses in photonics, electronics, and optics made PMMA the material of choice for the matrix. However, the use of PMMA is also important for the successful preparation of homogeneous phosphor mixtures homogeneously. This homogeneous distribution could result in more efficient light emission from the phosphors, and surface interactions could further enhance the emission intensity and light output efficiency. Ultimately, combining coordinated polymer materials with phosphor materials offers exciting opportunities for tailoring the optical properties to a variety of applications.

**Scheme 1:**
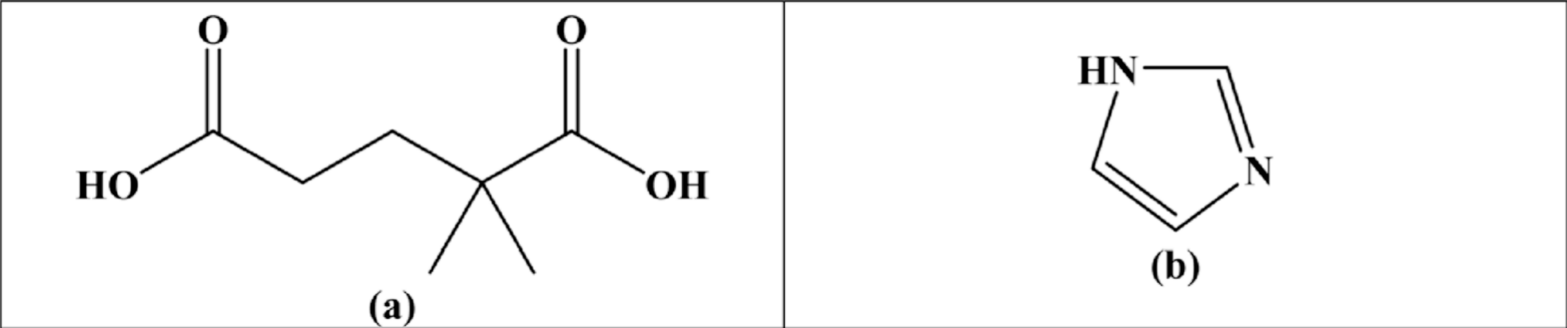
(a) 2,2-Dimethylglutaric Acid (dmgH_2_) and (b) Imidazole (im) Are the Structures of the Ligands^43^

In this study, a 3D metal–organic framework containing imidazole (im) and 2,2-dimethylglutarate (dmg^2–^) ligands {[Cu(μ_3_-dmg)(im)_2_]·3H_2_O}*_n_* (see Scheme 1) with phosphors (YAG:Ce, NCS:Sm, and CAO:Mn) was planned to improve optical and degradation properties. In order to clarify the effects and possible illumination records of CPs regarding YAG:Ce, NCS:Sm, and CAO:Mn phosphors, state-stable photoluminescence and decay time measurements were performed. In comparison to their nonadditive forms, the brightness of the YAG:Ce, NCS:Sm, and CAO:Mn phosphors blended with PKY159 in the PMMA matrix increased by roughly 29-, 5-, and 27-times, respectively. In order to clarify the process underlying the intensity enhancement, we recorded the decay curves on the microsecond time scale. When MOF particles are embedded in the PMMA in the proper ratio, they act as donors and phosphors as acceptors, according to experimental studies based on decay times and microsecond scale steady states. Particularly, in comparison to the additive-free versions, all of the phosphor composites with additive showed higher thermal stability, with activation energy values of 0.325, 0.265, and 0.236 eV for the phosphors YAG:Ce, NCS:Sm, and CAO:Mn, respectively, in the temperature range of 303–503 K. Coordinating bonds, electronic transitions, and synergistic effects of the CP units pave the way for enhanced luminescence output in phosphors, making these combined structures a promising candidate for applications in illumination, displays, and sensing units.

## 2 Experimental Section

### 2.1 Materials

Tetraethyl orthosilicate (TEOS, 98%), sodium nitrate (NaNO_3_), calcium nitrate tetrahydrate (Ca(NO_3_)_2_·4H_2_O, 99%), samarium nitrate tetrahydrate (Sm(NO_3_)_2_·4H_2_O, 99%), hydrochloric acid (HCl), yttrium(III) nitrate hexahydrate (Y(NO_3_)_3_·6H_2_O, 99.9%), aluminum nitrate nonahydrate (Al)NO_3_)_3_·9H_2_O, ≥98%), ethanol, cerium(III) nitrate hexahydrate (Ce(NO_3_)_3_·6H_2_O, ≥98%), acetic acid (CH_3_COOH), calcium nitrate tetrahydrate (Ca(NO_3_)_2_·4H_2_O, 99%), manganese nitrate tetrahydrate (Mn(NO_3_)_2_·4H_2_O, 99%), and acetone chemicals were of analytical grade and supplied from Sigma-Aldrich.

### 2.2 Instrumentation

Characterization of the synthesized powders is carried out by photoluminescence (PL), X-ray diffraction (XRD), X-ray photoelectron spectroscopy (XPS), Fourier transform infrared spectroscopy (FT-IR), and scanning electron microscopy (SEM) analysis. PL spectra were recorded using an Edinburgh Instruments FLS 920 spectrofluorometer equipped with a 450 W Xe lamp. Decay time data were measured using the same operating device according to the timing single photon counting (TCSPC) theory. Using a multichannel scaling card, periods of luminescence decay were measured with picosecond diode laser excitation at 467 nm or microsecond light lamp excitation at 460 nm. The emission and excitation half lengths were changed to 5.0 or 2.5 nm. The phase structure of the synthesized phosphors was examined with an X-ray diffractometer device (XRD, Thermo Scientific ARL X-ray diffractometer, Cu Kα, 1.5405 Å, 45 kV, 44 mA). For all samples, elemental composition and surface chemistry were determined by using X-ray photoelectron spectroscopy (XPS, Thermo Scientific K-Alpha) with a monochromatic Al–Kα (1486.7 eV) X-ray source and a 400 nm diameter beam size. Functional powder groups were evaluated by Fourier transform spectroscopy (FT-IR, Thermoscientific Nicolet I10). A Zeiss Gemini SEM 560 scanning electron microscope (SEM) was used for morphological characterization at different magnification rates.

### 2.3 Synthesis of MOF Particles

The synthesis and characterization of the {[Cu(μ_3_-dmg)(im)_2_]·3H_2_O}*_n_* coordination polymer were given in detail in our previous work.^43^ In a solution comprising imidazole, Cu(II) ions, and dmgH_2_, Compound 1 was synthesized. Initially, Cu(CH_3_COO)·2H_2_O dissolved in 25 mL of water was combined with dmgH_2_ drop by drop under stirring at 60 °C, resulting in the formation of a suspension. The mixture was then stirred for 5 h at the same temperature. Subsequently, imidazole was gradually added to the suspension. Following 2 h of stirring at 60 °C, the solution became clear and was allowed to cool to room temperature. Over 2 weeks, solvent evaporation led to the gradual formation of crystals. After filtration and washing with 10 mL of water, the crystals were air-dried to yield Compound 1.

### 2.4 Synthesis of Phosphor Materials

#### 2.4.1 YAG:Ce Phosphor

Ce^3+^-doped yttrium aluminum garnet (YAG:Ce) is a yellow phosphor widely used in optical display and lighting applications. According to the research conducted in the literature, Y_2–*x*_Al_5_O_12_:Ce_*x*_^3+^(Y_2.92_Al_5_O_12_:Ce_0.08_^3+^), which was synthesized by the sol–gel method containing 1% Ce^3+^, was weighed in the stoichiometric ratio of Y(NO_3_)_3_·6H_2_O, Al(NO_3_)_3_·9H_2_O and converted into ethanol. Then, Ce(NO_3_)_3_·6H_2_O dissolved in ethanol was added to the prepared solution and mixed at 60 °C. The pH value was adjusted to 1–2 with CH_3_COOH and kept at 80 °C for 24 h. Finally, it was calcined at 1000 °C for 2 h in air.^44^

#### 2.4.2 NCS:Sm Phosphor

Sm^3+^-doped sodium calcium silicate (Na_2_CaSiO_4_:Sm^3+^; NCS:Sm) is used as a potential pure orange emitting phosphor in white LED and other photonic device applications. According to the research conducted in the literature, for Na_2_Ca_1–*x*_SiO_4_:Sm_*x*_^3+^ (Na_2_Ca_0.9_SiO_4_:Sm_0.1_^3+^), which was synthesized by the sol–gel method containing 1% Sm^3+^, first 50 mL of TEOS, 50 mL of ethanol, and 10 mL of deionized water were added in stoichiometric ratios at 65 °C and stirred for 1 h. NaNO_3_, Ca(NO_3_)_2_·4H_2_O, and Sm(NO_3_)_2_·4H_2_O, which were dissolved separately in water, were added onto TEOS and mixed. The pH was adjusted to 1 by adding HCl to the final solution. The resulting gel was kept at 65 °C for 3 h. It was dried at 90 °C for 18 and 5 h at 110 °C. It was then calcined at 1100 °C for 3 h in air.^13^

#### 2.4.3 CAO:Mn Phosphor

CaAl_12_O_19_:Mn^4+^ (CAO:Mn) red phosphor particles were synthesized in this work by using the sol–gel method. A detailed description of the applied sol–gel technique was shared in our previous study.^45^ In order to synthesize related phosphor, precursor materials such Ca(NO_3_)_2_·4H_2_O, Al(NO_3_)_3_·9H_2_O, and Mn(NO_3_)_2_·4H_2_O were utilized and dissolved in ethanol. The process of obtaining the particles involved: mixing, drying, and calcining at 1200 °C for 4 h in air. The greatest PL intensity value of 1.0% (%w/w) was found when the emission-excitation spectra were considered for all three concentrations. Therefore, this concentration value is determined in all further investigations.

### 2.5 Characterizations of YAG:Ce, NCS:Sm, and CAO:Mn Particles

#### 2.5.1 YAG:Ce

**Figure 1:**
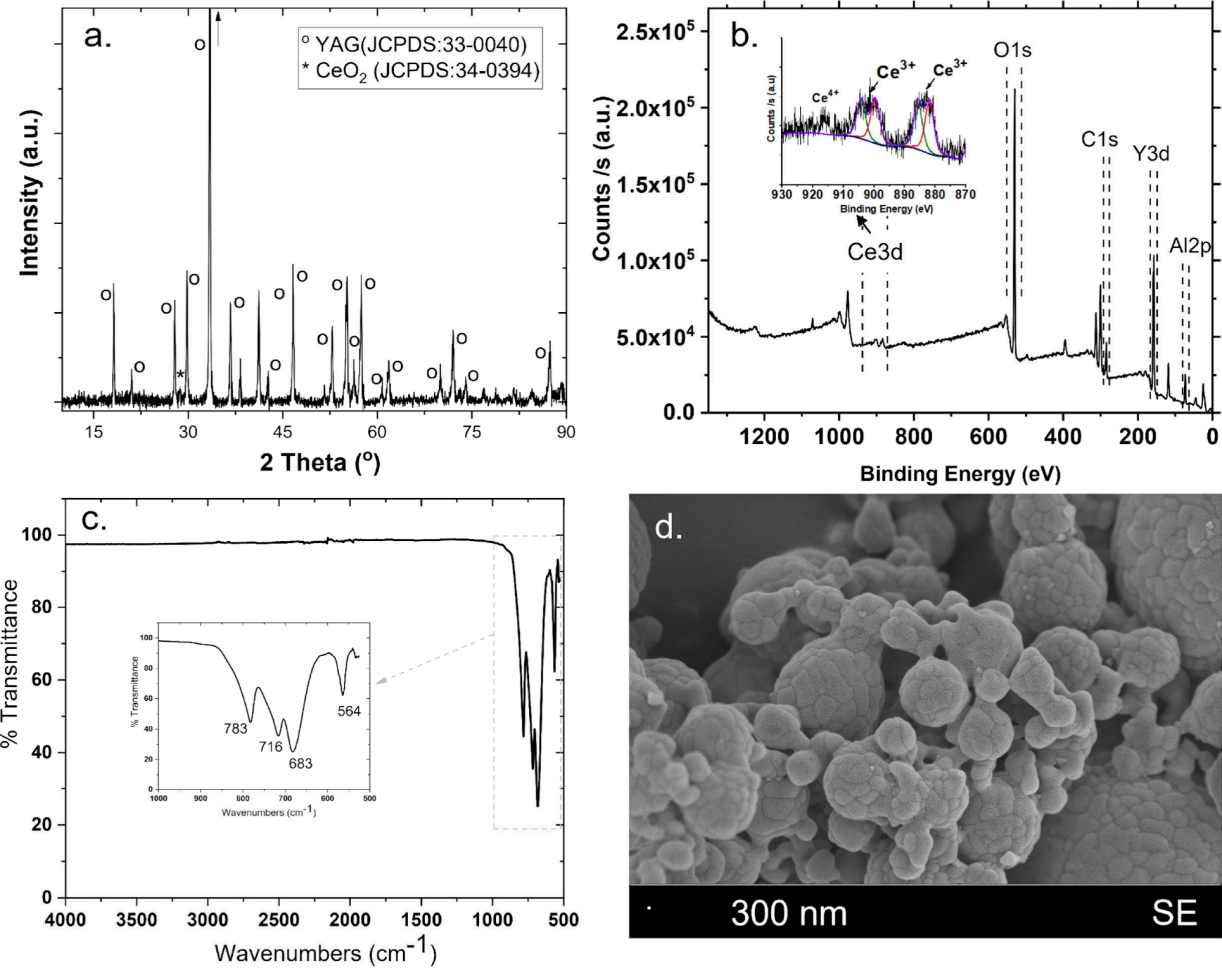
(a) XRD pattern, (b) XPS spectrum, (c) FTIR spectrum, and (d) SEM micrograph of the YAG:Ce powders.

Figure 1 (a) shows the XRD pattern of YAG:Ce phosphor particles. The main diffraction peaks at 18.30, 27.80, 29.50, 33.20, 36.30, 41.30, 46.80, 55.20, 57.60, 61.69, 72.01, and 87.38 correspond to YAG structure (JCPDS card no. 33–0040).^46^ Results indicate that a minor peak at the 28.7 position, which corresponds to the formation of the CeO_2_ phase as well (JCPDS No. 34–0394), was observed.^47^ The XPS spectra for the sol–gel-synthesized YAG:Ce sample are displayed in Figure 1 (b). The YAG:Ce structure is verified by the binding energy values, which were obtained for O 1s (530.43 eV), C 1s (284.95 eV), Al 2p (73.86 eV), Y 3d (158.11 eV), and Ce 3d (883.81 eV). The fitting results where two distinct peaks are seen for Ce^3+^ show that four lines with respective centers at 881.82, 885.57, 889.60, and 904.15 eV dominate the overall Ce 3d emission. These figures are in line with Ce^3+^ data that have already been issued. Only the minor peak around 915 eV leads to the presence of CeO_2_ inclusions. From the data obtained, it can be interpreted that Ce^4+^ ions in the synthesized YAG:Ce phosphor are almost completely reduced to Ce^3+^ ions, according to the comparison with the XPS spectra of Ce^4+^ for CeO_2_.^48^ The range of the FTIR spectra of the YAG:Ce phosphor powders was between 500 and 4000 cm^–1^ (see Figure 1 (c)). The features of the Y–O (metal–oxygen) vibrations of the YAG structure are represented by the peaks at 683 and 564 cm^–1^, whereas the peaks at 783 and 716 cm^–1^ can be attributed to Al–O (metal–oxygen) vibrations. The YAG structure formed as a result of a distinct separation of the Y–O and Al–O peaks, and these findings are in line with the XRD findings.^49^ It is evident from the SEM images (see Figure 1 (d)) that small particles recombine to form bigger aggregates which might be due to interfacial attractions and the calcination.^50^

#### 2.5.2 NCS:Sm

**Figure 2:**
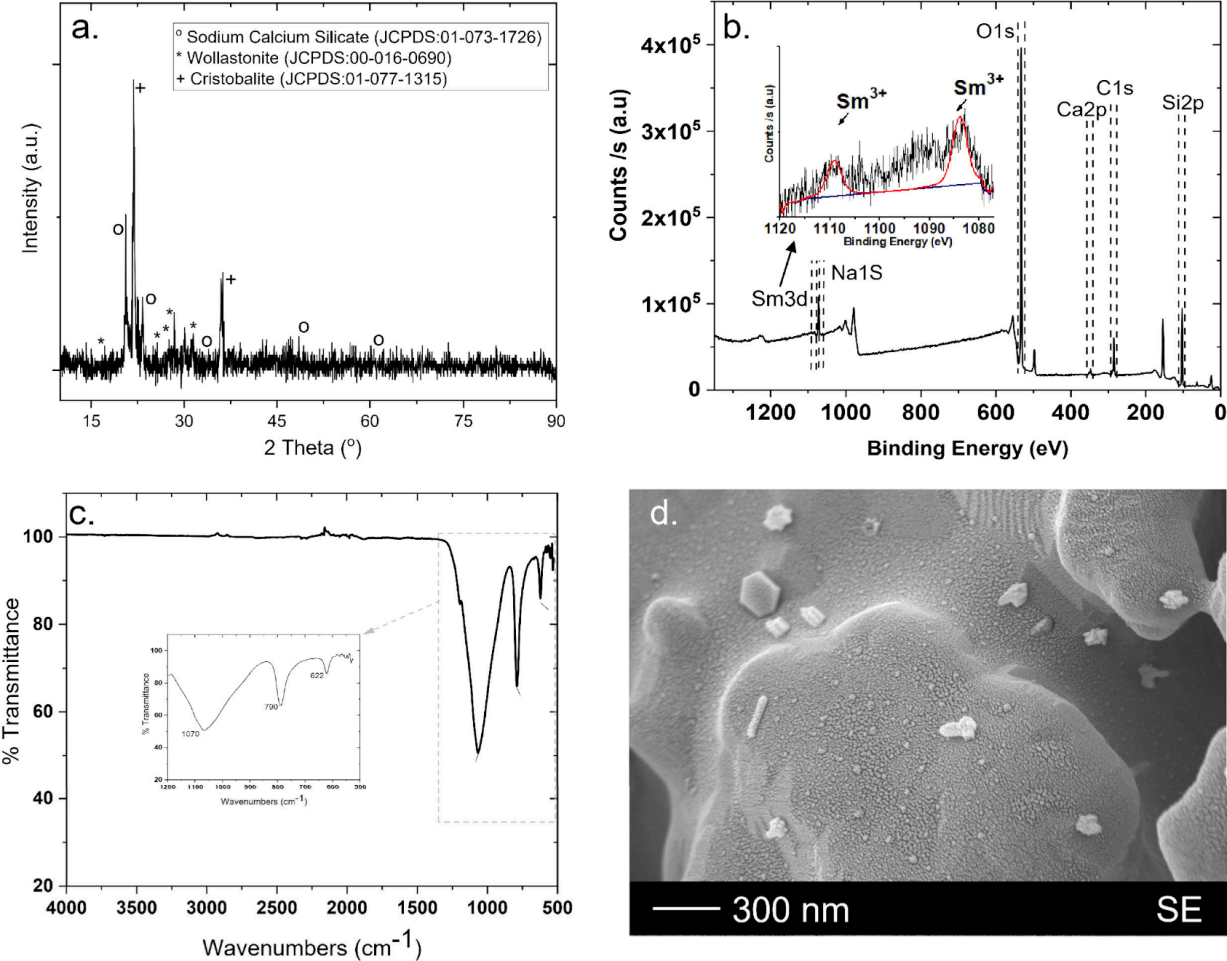
(a) XRD pattern, (b) XPS spectrum, (c) FT-IR spectrum, and (d) SEM micrograph of the NCS:Sm powders.

Figure 2 (a) shows the XRD diffraction pattern of NCS:Sm phosphor particles. According to the pattern it can be inferred that, in addition to the sodium calcium silicate (Na_2_CaSiO_4_, JCPDS: 01–073–1726) phase, two additional phases were obtained as another sodium calcium silicate compound (Na_2_Ca_3_Si_6_O_16_, JCPDS: 00–016–0690) wollastonite and cristobalite (SiO_2_, JCPDS: 01–077–1315).^51^ However, any phases related to Sm compounds cannot be observed in the patterns, showing the successful incorporation of Sm into the structure. Figure 2 (b) shows the XPS spectra of the sol–gel-produced NCS:Sm sample. The NCS:Sm structure is verified by the binding energy values, which were 532.34 eV for the O 1s, 285.03 eV for the C 1s, 103.15 eV for the Si 2p, 1071.98 eV for the Na 1s, 347.25 eV for the Ca 2p, and 1082.97 eV for the Sm 3d. It also shows two distinct peaks at 1082.97 and 1109.20 eV, respectively, which correspond to Sm 3d5/2 and Sm 3d3/2. The +3 oxidation state of Sm in this host is confirmed by the energy difference of 26.23 eV between the two peaks.^52^ FTIR spectra of NCS:Sm phosphor powder were recorded in the range of 500–4000 cm^–1^ (see Figure 2 (c)). The characteristic bands located at 1100–780 cm^–1^ can be attributed to various stretching modes of Si–O and the metal–oxygen (Ca–O) bond vibration in the band located at ∼620 cm^–1^. A glassy morphology (see Figure 2 (d)), due to the aforementioned phases in the XRD part, was obtained where partially crystalline parts are observed in the images.^13^

#### 2.5.3 CAO:Mn

**Figure 3:**
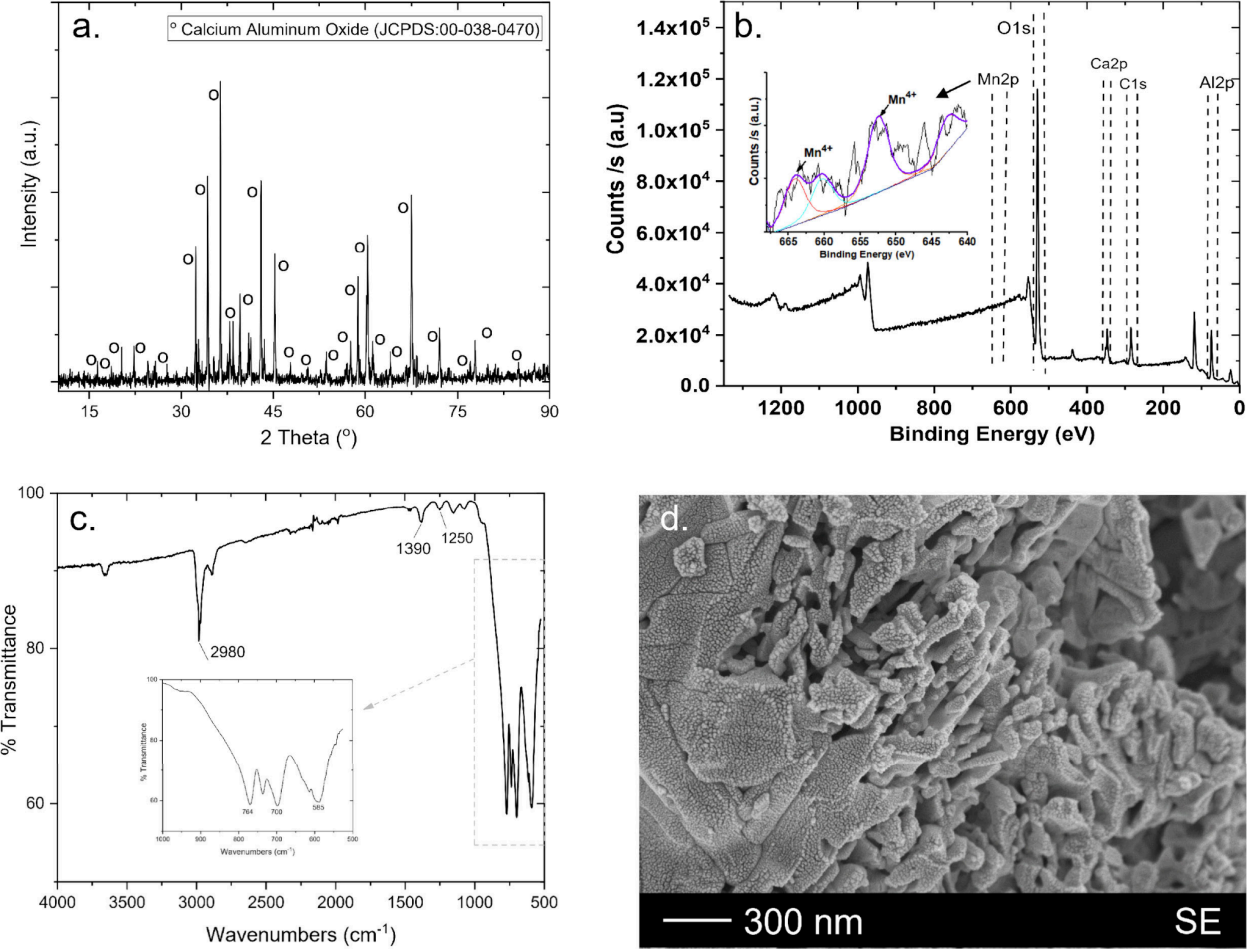
(a) XRD pattern, (b) XPS spectrum, (c) FTIR spectrum, and (d) SEM micrograph of the CAO:Mn powders.^45^

Figure 3 (a) depicts the powder CAO:Mn phosphor’s XRD diffraction pattern. According to the pattern, it can be expressed that a single phase, calcium aluminum oxide (CaAl_12_O_19_ JCPDS: 00–038–0470), was obtained without any Mn compound based phases. The obtained results together with the XPS results confirm the Mn incorporation into the structure. Figure 3 (b) shows the XPS spectra for the CAO:Mn sample that was synthesized by using the sol–gel method. The CAO:Mn structure is confirmed by the binding energy values, which were found to be around 529 eV for the O 1s, 284 eV for the C 1s, 347 eV for the Ca 2p, 74 eV for the Al 2p, and 641 eV for the Mn 2p. Figure 3 (b) also displays the Mn 2p core-level XPS spectra and fitted lines to investigate the manganese ions’ valence state. The presence of Mn^4+^, which appears in the spectrum as 2p1/2 and 2p3/2 of Mn^3+^ and Mn^4+^, is confirmed by the peaks at 650 and 665 eV.^53^ FTIR spectroscopy was used to analyze the functional groups that were present in the powder that was calcined at 1200 °C during the crystallization of the produced powders (Figure 3 (c)). The spectra’s peaks at around 764 and 585 cm^–1^, respectively, represent the stretching and bending modes of AlO_6_. C–H stretching bonds were identified by assigning peaks between 2850 and 3000 cm^–1^ to them. The fact that there is not a peak between 1250 and 1650 cm^–1^ suggests that nitrates were lost from the precursor materials throughout the process of decomposition. It is evident from the SEM images that the particles are not homogeneous and are mostly aggregated during the 1200 °C calcination process (see Figure 3 (d)).^45^

### 2.6 Preparation of Composites

The composites were prepared by combining PMMA (120 mg), phosphors (5 mg), and an equivalent mass of the {[Cu(μ_3_-dmg)(im)_2_]·3H_2_O}*_n_* complex (PKY159) in THF solvent with constant mixing by the magnetic stirrer. This viscous mixture was coated onto a 125 μm Mylar (TM type) substrate using a spin coating device. Movies recorded using the Ambios Technology XP-2 Surface Profilometer had outputs of 4.36 ± 0.10 μm (*n* = 14). After cutting to the appropriate size, the thin films were placed diagonally in the Quartz cuvette or solid sample holder of the instrument. Table 2 shows the composition ratios of the additives used.

**Table 2:** Composition of the Exploited Materials and Additives

Phosphor (5 mg)	Matrix (120 mg)	Additive (5 mg)	Solvent (2 mL)
YAG:Ce	PMMA	-	THF
YAG:Ce	PMMA	PKY159	THF
NCS:Sm	PMMA	-	THF
NCS:Sm	PMMA	PKY159	THF
CAO:Mn	PMMA	-	THF
CAO:Mn	PMMA	PKY159	THF

## 3 Results and Discussion

To evaluate in detail the interaction between green, orange-red, and red phosphors and the coordination polymers used as additives, the spectral properties of the phosphors were investigated in two different configurations in the presence and absence of PKY159 in the polymeric matrix. The coordination polymer contains a copper (Cu) center compatible with {[Cu(μ_3_-dmg)(im)_2_]·3H_2_O}_*n*_, 2,2-dimethylglutarate (dmg^2–^) ligands, and imidazole (im) ligands. When excited at 380 nm, the emission peaks observed at 430 and 485 nm probably consist of specific electronic transitions resulting from copper center-ligand interactions within the complexes. Also, its large surface area and porous structure enable interactions with the surface of the phosphor particles. The coordination of the copper center with dmg^2–^ and imidazole ligands contributes to its unique electronic configuration, which enhances its optical properties. Specifically, the electron-donating or -withdrawing nature of these ligands significantly influences the energy levels and transition probabilities, resulting in distinct emission peaks observed at 430 and 485 nm. The proximity of the copper centers and imidazole groups may contribute to the broadening or strengthening of the phosphors’ emission spectra.

**Figure 4:**
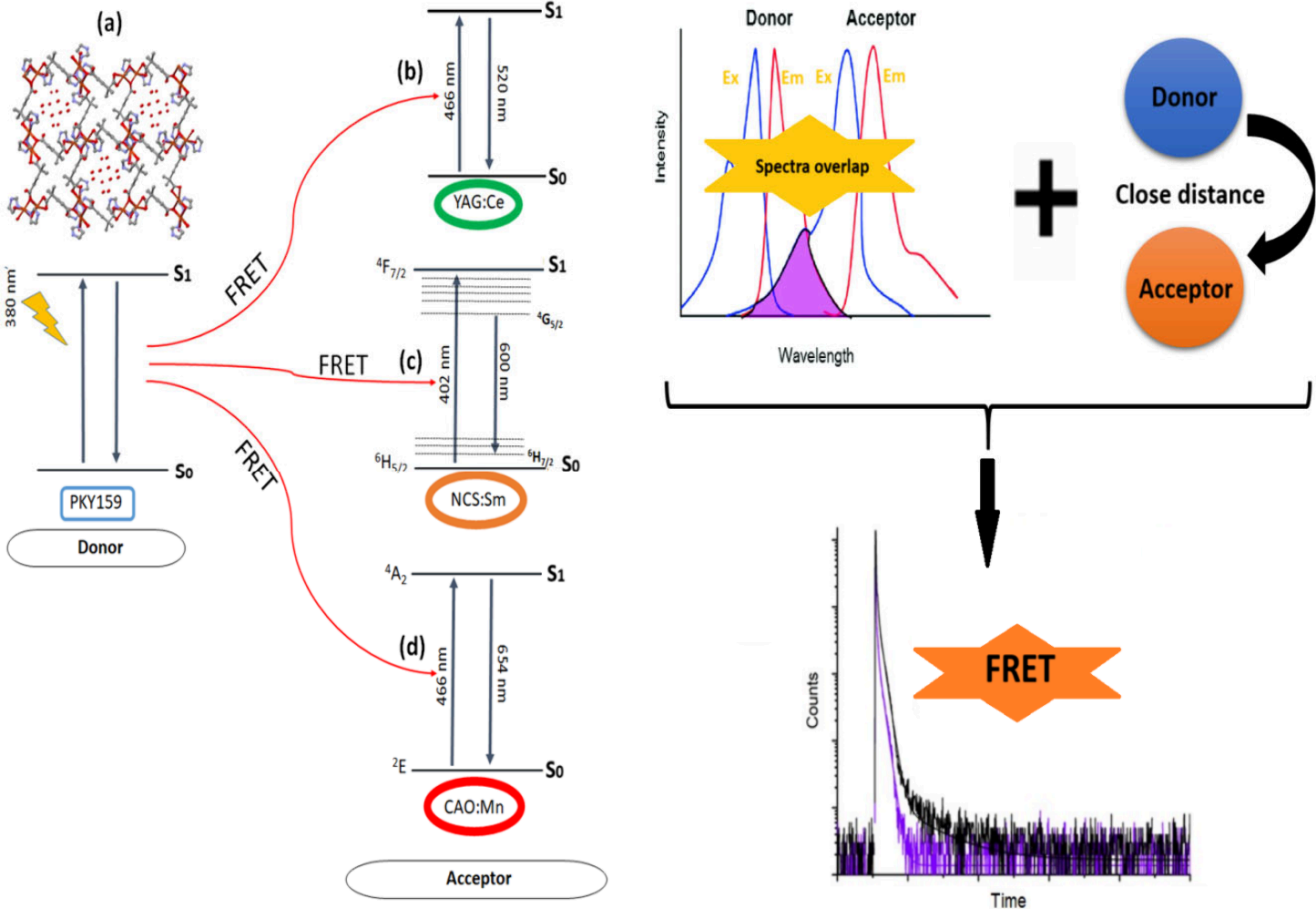
Possible energy transfer paths between the MOF and phosphors: (a) PKY159, (b) YAG:Ce/PKY159, (c) NCS:Sm/PKY159, and (d) CAO:Mn/PKY159 in PMMA.

The 380 nm excitation probably amplifies the electrons to higher energy levels within the fundamental energy complex. When these adapted electrons return to lower energy levels, they emit photons corresponding to specific wavelengths, resulting in the observed emission peaks (Figure 4 (a)). When combined with phosphor materials, the three-dimensional (3D) network of PKY159 enables efficient energy transfer. This structure optimizes energy flow, leading to more efficient photon conversion and thus improved light emission. The structural features and electronic configuration of the PKY159 complex determine the precise nature of these transitions. For example, the electron-donating or -withdrawing capacity of the ligands and the geometry around the copper center affect the energy generation and transition probabilities. To fully determine the structural state that causes these transitions, ligand field effects, the electronic configuration of the metal center, its coordinated geometry and complexation, as well as other electronic interactions need to be followed. During this research, it was investigated which species can spend energy. The emission bands of PKY159 and the first excitation band of the phosphors essentially overlap. This overlap is strong evidence that energy transfer can occur. Detailed examination of the spectrum reveals suggestive evidence that a fluorescence resonance energy transfer (FRET) of phosphors and strain PKY159 may be involved. The polymer materials were arranged with phosphor materials as donors and acceptors, the energy dissipation rates of which involve FRET. The most widely used mechanism for fluorescence sensing is FRET, a distance-dependent nonradiative energy transfer process. When the emission spectrum of the donor partially overlaps the absorption spectrum of the acceptor, energy is transferred from the donor to the acceptor through the FRET process. The efficiency of FRET depends on a number of factors, such as the extent of the spectral overlap, the distance between the donor and the acceptor, and the dipole–dipole interaction. The interaction mechanism between PKY159 and YAG:Ce, NCS:Sm, and CAO:Mn phosphors used in this study is attempted to be explained in Figure 4 (a), (b), (c), and (d). The {[Cu(μ_3_-dmg)(im)_2_]·3H_2_O}*_n_* structure itself might have light absorption capabilities, which could enhance the emission properties of the phosphors. By providing an additional pathway for light absorption, the MOF could contribute to the overall photoluminescence performance of the composite material. Also a detailed review of energy transfer in which coordination polymer materials serve as donors and phosphor materials as acceptors is discussed below.

### 3.1 Spectral Properties of the YAG:Ce/PKY159 Binary Blends

**Figure 5:**
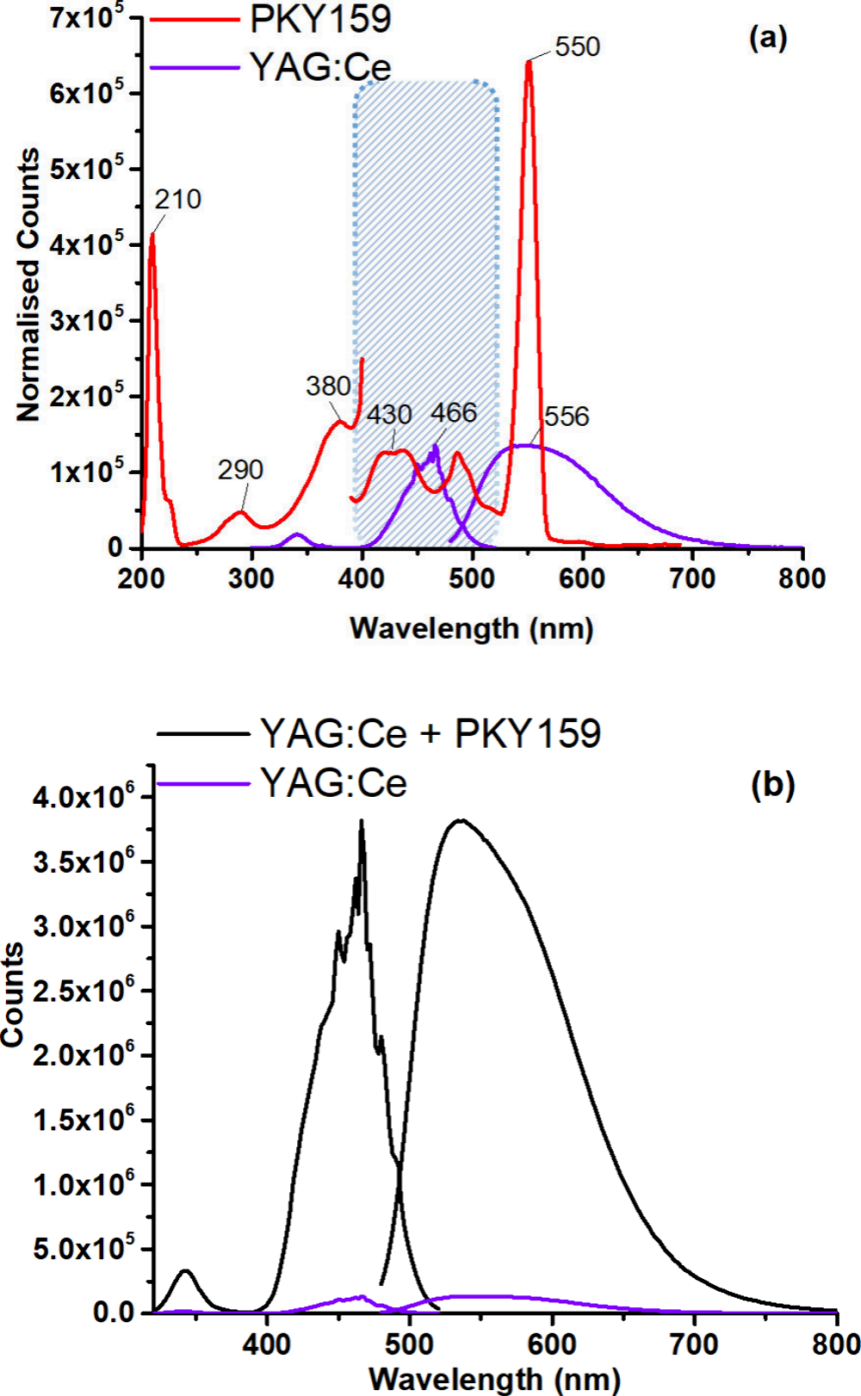
(a) Individual luminescence spectra normalized for PKY159 and YAG:Ce in the PMMA thin film. (b) Excitation/emission spectra of the additive-free YAG:Ce and PKY159 blended forms (λ_max_^ex^: 466 nm).

The emission and excitation spectra of YAG:Ce when integrated into the PMMA polymer matrix were analyzed in two different scenarios: without and with PKY159 contribution. The dispersion-based excitation spectrum of YAG:Ce at 466 nm wavelength is shown in Figure 5 (a). The adaptation of electrons from the ground state of the 4f1 electron configuration to the 5d1 enlargement of Ce^3+^ ions is responsible for the two distinct peaks in its excitation. The spectra were observed at 340 and 466 nm, respectively. These peaks represent energy thresholds at which electron transitions in the cerium ions can begin. Moreover, the emission spectrum of YAG:Ce generally shows a large peak in the blue-green part of the spectrum, centered at 520 nm. The 5d–4f transitions in Ce^3+^ ions are the competitiveness of this emission.^54^ To evaluate the interactions behind the increase in the intensity of the phosphors, we recorded the optical spectra of PKY159 and YAG:Ce separately at their respective excitation wavelengths. The emission band of PKY159 and the excitation band of YAG:Ce overlap in the figure. The mechanism involving FRET enables efficient nonradiative energy transfer from the donor (coordination polymer) to the acceptor (phosphor material) and enables enhanced luminescence properties in optoelectronic applications. Thus, it is thought that the energy transferred from the donor complex to the acceptor phosphors effectively excites the Ce^3+^ ions in the YAG cage. After adaptation, Ce^3+^ ions undergo characteristic 5d–4f transitions, resulting in photon emission at 540 nm. This emission contributes to the increase in the photoluminescence intensity observed when the two materials are mixed.

Moreover, the comparative absorption and emission spectra of YAG:Ce at 466 nm in the presence and absence of the PKY159 dopant are shown in Figure 5 (b). Compared to its free form, a 29-fold (97%) increase in the intensity of YAG:Ce was observed in the presence of the PKY159 additive (see Table 3).

**Table 3:** *I*/*I*_0_ Parameters of the Utilized Composites^a^

Phosphor + Additive	Excitation (nm)	Emission (nm)	Intensity Fold (*I*/*I*_0_)
YAG:Ce + PKY159	460	540	28.68
NCS:Sm + PKY159	402	602	4.80
CAO:Mn + PKY159	466	654	27.03

a *I*_0_ = emission intensity of additive-free phosphor; *I* = emission intensity of phosphor + PKY159 blend form.

### 3.2 Spectral Behavior of the NCS:Sm/PKY159 Binary Blends

**Figure 6:**
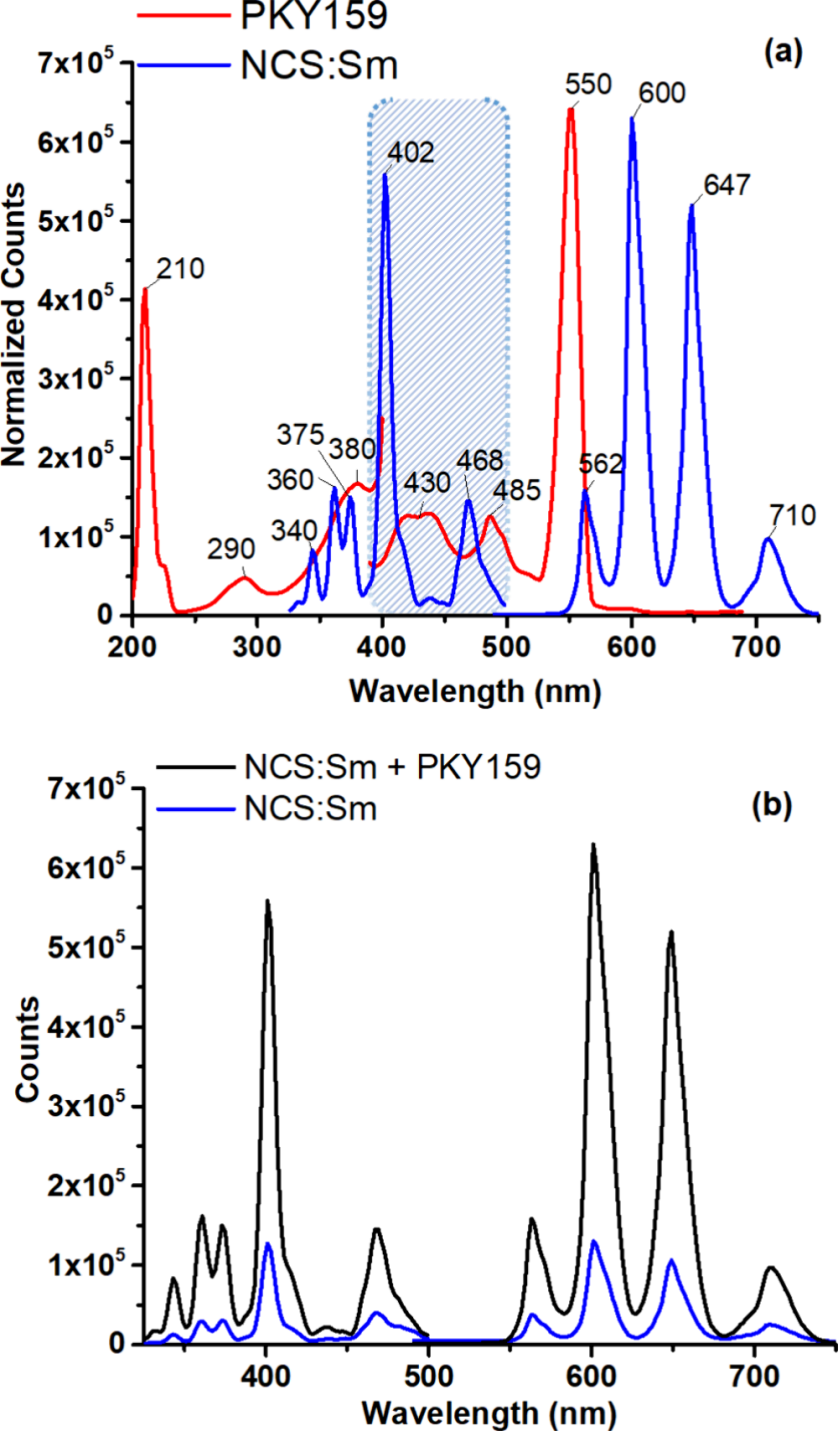
(a) Individual luminescence spectra normalized for PKY159 and NCS:Sm in the PMMA thin film. (b) Excitation/emission spectra of the additive-free NCS:Sm and PKY159 blended forms (λ_max_^ex^: 402 nm).

Figure 6 (a) shows the individual excitation and emission spectra of the NCS:Sm phosphors and the PKY159 dopant. When the catalyst is excited at 402 nm, the maximum emission wavelength is observed at 600 nm. It shows several sharp bands in the excitation spectrum at 340, 360, 375, 402, 416, 436, and 467 nm. These range from the ground state ^6^H_5/2_ to different excited states such as ^4^H_9/2_, ^4^D_3/2_, ^4^D_1/2_, ^4^F_7/2_, ^4^ M_19/2_, ^4^G_9/2_, and (^4^I_11/2_ + ^4^I_13/2_ + ^4^M_15/2_), respectively. It is attributed to f–f transitions. The emission spectrum for the NCS:Sm phosphor also exhibits three distinct peaks located at 565, 600, and 647 nm due to characteristic transitions, which can be attributed to the ^4^G_5/2_ → ^6^H_5/2_, ^4^G_5/2_ → ^6^H_7/2_, and ^4^G_5/2_ → ^6^H_9/2_ transitions, respectively.^13^

The process begins with the absorption of photons by the polymer material, which is jointly coordinated by its donors. This absorption is maintained by adapting the electrons within the coordinating polymer to higher energy states. When the donor (tailored coordination polymer) and acceptor (phosphor material) are in proximity within a certain nanometer range, FRET occurs. The energy from the transmitter is transferred nonradiatively to the receiver through dipole–dipole interactions or other resonance energy distribution. The energy received by phosphor materials raises its electrons to higher energy states. When these electrons return to their ground state, they release energy into the photons, causing luminescence. The emission spectrum of the receiver is observed, and this process reveals the overall luminescence properties of the combined system.

When excited at 402 nm, Figure 6 (b) compares the absorption and emission spectra of the additive-free and PKY159 additives containing NCS:Sm, respectively. In comparison to the free form, the phosphor-coordination polymer blend showed a 5-fold (79%) (see Table 3) increase in intensity-based measurements.

### 3.3 Spectral Behavior of the CAO:Mn/PKY159 Binary Blends

**Figure 7:**
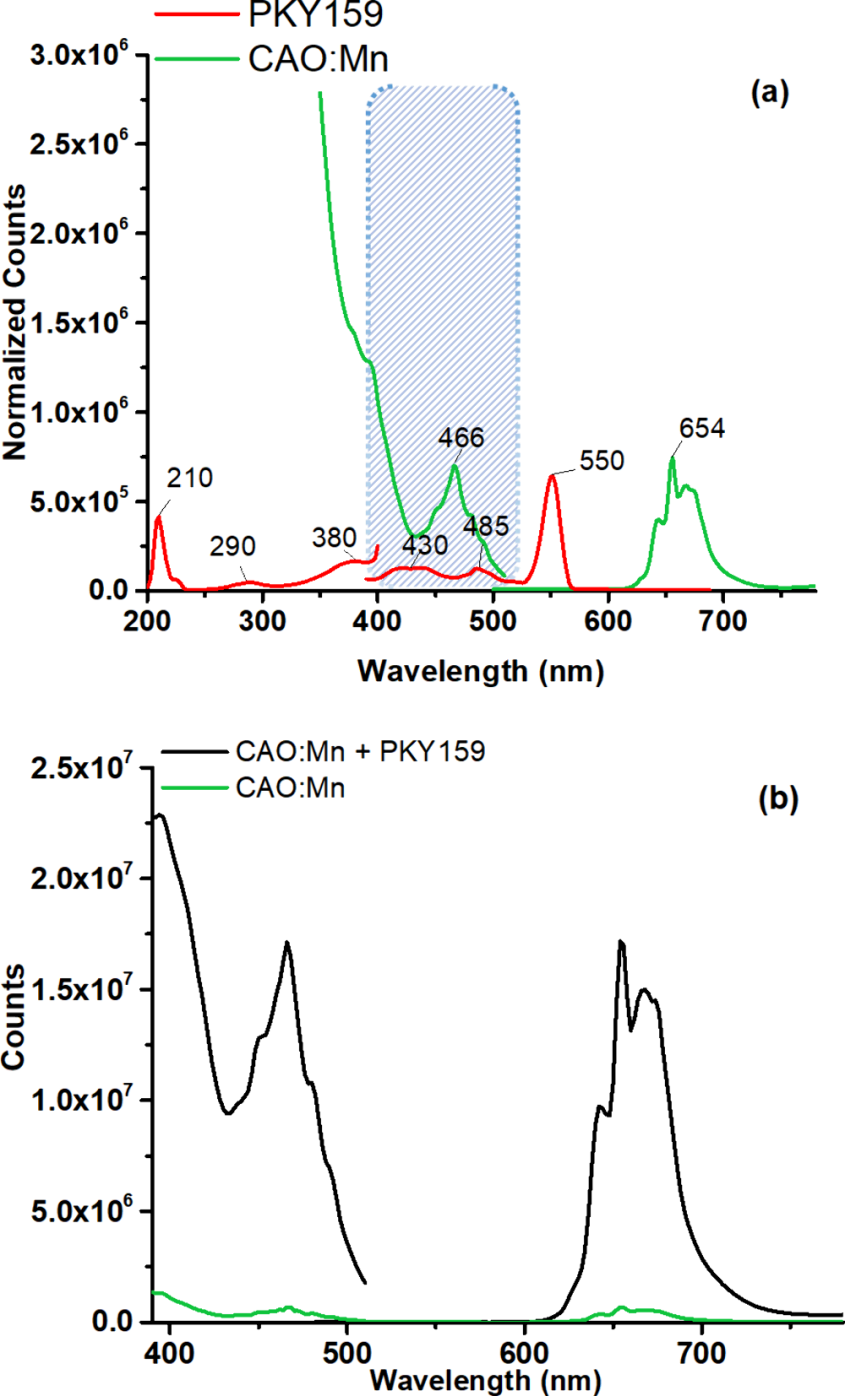
(a) Individual luminescence spectra normalized for PKY159 and CAO:Mn in the PMMA thin film. (b) Excitation/emission spectra of the additive-free CAO:Mn and PKY159 blended forms (λ_max_^ex^: 466 nm).

The absorption and emission spectra of CAO:Mn phosphors are shown in Figure 7 (a). Based on excitation at a wavelength of 466 nm, the emission spectrum shows a narrow band from 600 to 750 nm with three sharp peaks at approximately 643, 654, and 666 nm due to the transition of Mn^4+^ from ^2^E to ^4^A_2_. However, the CAO:Mn absorption spectrum shows a broad band from 280 to 520 nm, with three peaks at roughly 330, 398, and 466 nm.^45^

Red phosphors exhibited an increase of 27.03 (*I*/*I*_0_) (see Table 3) in both absorption and emission intensity when combined with PKY159 particles (see Figure 7 (b)). The first excitation band of CAO:Mn largely overlaps with the emission bands of PKY159 nanoparticles (Figure 7 (a)). A large overlap indicates a high probability of energy transition. Careful examination of both spectra shows evidence of FRET potential between CAO:Mn and coordination polymer particles. The unexpected increase in the emission-based intensity of red phosphors compared to other phosphors can be explained by selecting FRET pairs that offer a large degree of separation between emission peaks but also have an amount of spectral overlap.

### 3.4 Influence of {[Cu(μ_3_-dmg)(im)_2_]·3H_2_O}*_n_* (PKY159) Particles on Synthesized Phosphor Luminescence Characteristics

**Figure 8:**
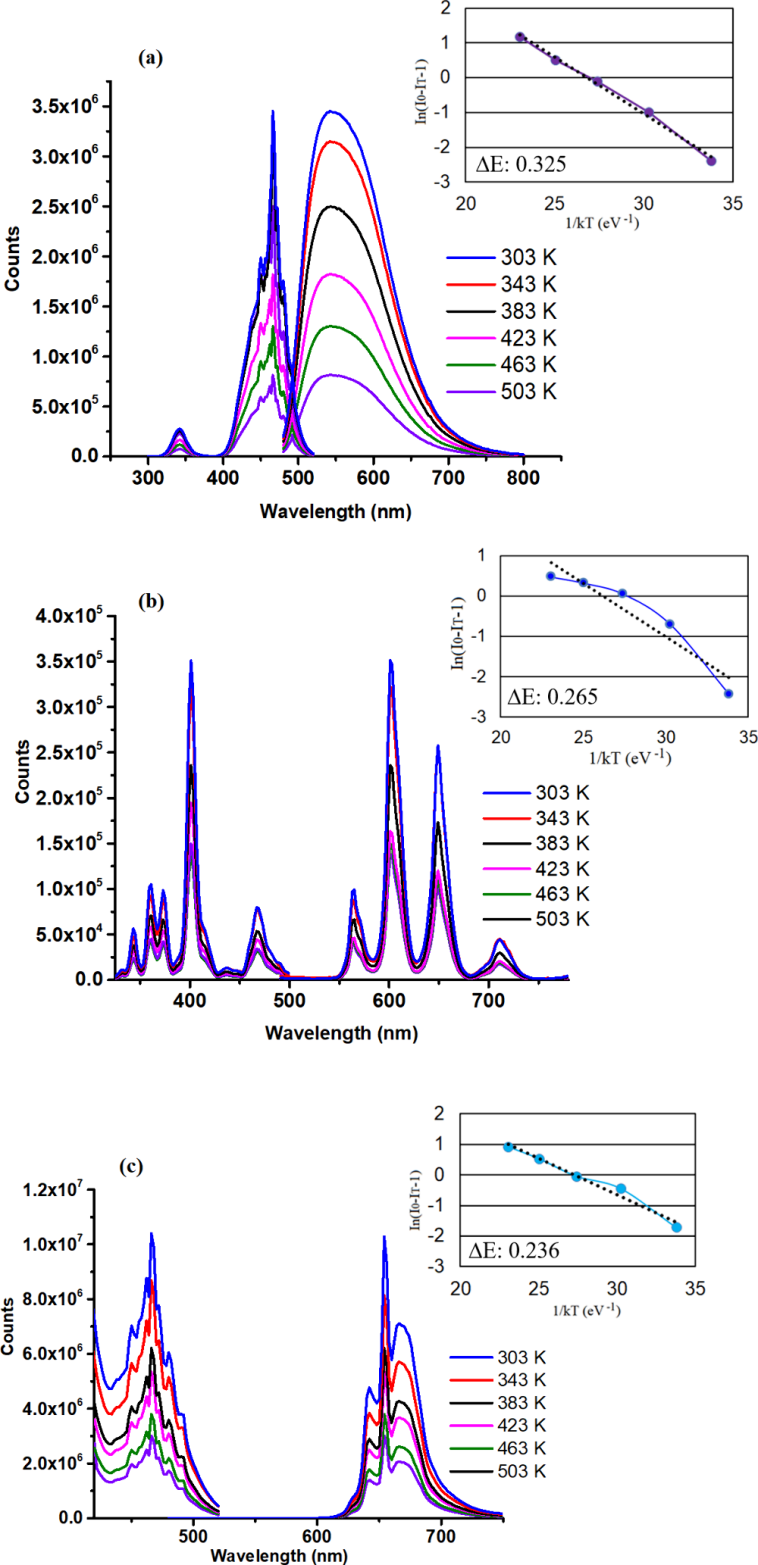
Emission spectra of PKY159 mixed forms with phosphors of (a) YAG:Ce, (b) NCS:Sm, and (c) CAO:Mn, depending on the temperature in the range of 303–503 K. Insets: Plots of the activation energy, Δ*E*, for YAG:Ce, NCS:Sm, and CAO:Mn phosphors in the presence of PKY159 are displayed as ln(*I*_0_/*I*_T_ – 1) versus 1/*kT*.

Research on luminescence thermal quenching is essential because it significantly influences the efficiency and stability of phosphor materials used in high-temperature applications, including solid-state lighting and imaging technologies.^13,55^ In this study, the photoluminescence (PL) properties of YAG:Ce, NCS:Sm, and CAO:Mn phosphor materials were investigated by incorporating coordination polymer PKY159. A variety of temperature treatments, including 303, 343, 383, 423, 463, and 503 K, were applied to the composite material. Every temperature step’s PL emission spectra were captured, showing an ongoing decrease in emission strength as the temperature increased (see Figure 8 (a), (b), and (c)). Thermal quenching is the cause of the observed decrease in emission intensity. When nonradiative relaxation processes become activated by thermal energy at high temperatures, thermal quenching occurs. The total emission intensity decreases as a result of these processes competing with radiative recombination.^56,57^

Additionally, the activation energy can be calculated using the Arrhenius equation below to gain a better understanding of the thermal quenching features.^58^
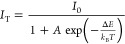
1Here *I*_0_ and *I*_T_ are the photoluminescence intensity at room temperature and *T* (K) the temperature, respectively. Δ*E*, *k*_B_, and *C* are the activation energy, Boltzmann’s (8.67 × 10^–5^ eV/K), and arbitrary constant, respectively. From the slope of the linear fitted graph between ln[(*I*_0_/*I*_T_) – 1] and (1/*k*_B_*T*), the value of activation energy for YAG:Ce along with PKY159 is recorded to be 0.325 eV which is higher than the commercial YAG:Ce phosphor (∼0.3 eV) (see Figure 8 (a) and inset).^59^ The activation energy of NCS:Sm with the MOF additive was determined to be 0.265 eV based on the good fitting of the experimental data into a linear line with a slope of −0.265 (see Figure 8 (b) and inset). Furthermore, it was observed that the activated energy of the synthesized NCS:Sm phosphor was around 0.283 eV, which was in close agreement with the results reported in the literature.^13^ Additionally, the activation energy of ∼0.236 eV was determined for the thermal quenching of CaAl_12_O_19_:Mn^4+^ with a PKY159 additive, which was greater than that of the nonadditive form of red-phosphor (*E*_a_ = ∼0.22 eV) reported in the literature.^8^ As a result, the experimental result shows that the related phosphors with the MOF addition have relatively good thermal stability and outstanding temperature-dependent luminous capabilities. The greater activation energy value of the synthesized phosphor suggests that it is more thermally stable, which increases its potential application as an orange-red, red, and green light-emitting component in white LEDs.

**Figure 9:**
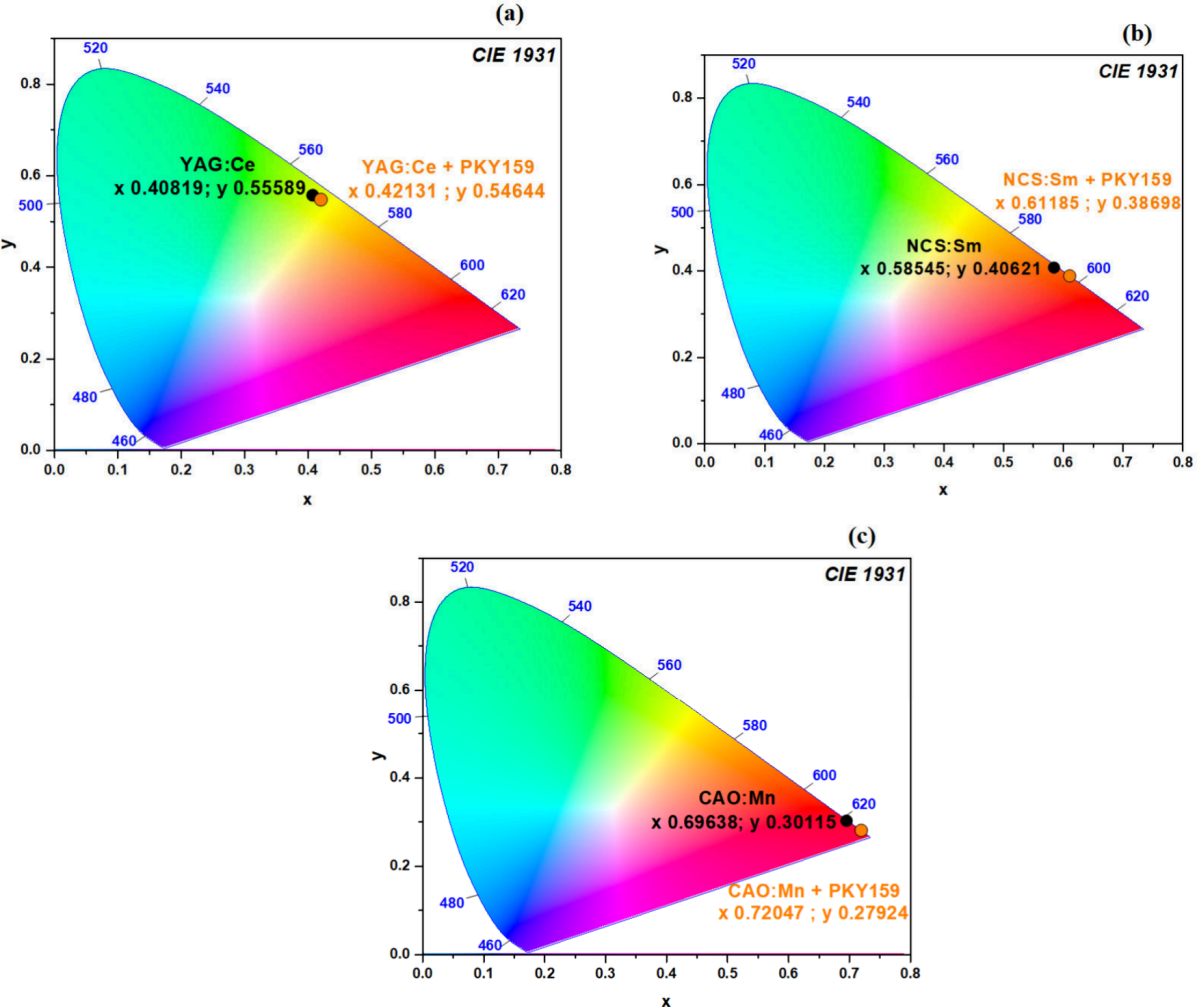
Color coordinates for phosphors (a) YAG:Ce, (b) NCS:Sm, and (c) CAO:Mn in the CIE 1931 chromaticity diagram in both the presence and absence of the PKY159 MOF additive.

The International Commission edeL’Eclairage (CIE) 1931 chromaticity diagrams of all three phosphors without and with additive versions are displayed in Figure 9 (a), (b), and (c). Table 4 shows the color coordinates for this diagram. The color coordinate values for free from YAG:Ce were (0.40819, 0.55589), as can be observed in Figure 9 (a). However, these values were changed to (0.42131, and 0.54644) after PKY159 was added. Similarly, it was discovered that the undoped values for NCS:Sm and CAO:Mn were (0.58545, 0.40621) and (0.69638, 0.30115), respectively. These values were for both phosphors (0.61185, 0.38698) and (0.72047, 0.27924) with the addition of PKY159. As indicated by the intersections of the *x* and *y* coordinates close to the edge of the CIE chromaticity map, all phosphors with additives exhibit a high degree of color purities according to these data.

**Table 4:** Particular CIE Chromaticity Coordinates and Internal Quantum Efficiencies for Each Phosphor and Each Combination of Phosphors with PKY159

Phosphor	CIE Coordinates	IQE (%)
YAG:Ce	(0.40819, 0.55589)	86.3
YAG:Ce + PKY159	(0.42131, 0.54644)	95.6
NCS:Sm	(0.58545, 0.40621)	60.1
NCS:Sm + PKY159	(0.61185, 0.38698)	66.3
CAO:Mn	(0.69638, 0.30115)	62.3
CAO:Mn + PKY159	(0.72047, 0.27924)	84.6

An important measure in optoelectronics and photonics, internal quantum efficiency (IQE), indicates how well photons are produced inside a material or apparatus due to electron–hole recombination. It can be defined as the ratio of photons produced to injected electron–hole pairs. The IQE values of the phosphors synthesized in this study, measured using the integrating sphere method, are reported in Table 4.

The following formula was used to calculate the IQEs:^60,61^
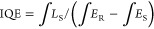
2where *L*_S_ is the phosphor’s emission spectrum; *E*_S_ and *E*_R_ are its excitation spectra; and BaSO_4_ is used as a reference in the integrating sphere, respectively. When PKY159 blend forms were compared with the additive-free forms of phosphors, an improvement in IQE values was recorded simultaneously with an increase in emission-based concentrations. This improvement can be attributed to the incorporation of phosphors into the MOF additive. According to calculations, the IQE of the YAG:Ce phosphor composite blends containing PKY159 was 95.6% at 466 nm excitation. Similarly, in the presence of PKY159, the internal quantum efficiency of NCS:Sm increased from 66.3% compared to that of the similar free form. The IQE of CAO:Mn along with the additive reached 84.6% (see Table 4). According to these findings, these materials show promise for high-efficiency lighting and display technologies, as these high IQE values show that they can improve optoelectronic device performance.^8,39,62^

### 3.5 Decay Time Measurements

Metal–organic coordination polymers have emerged as a fascinating class of materials due to their diverse structures and intriguing properties. Among these, {[Cu(μ_3_-dmg)(im)_2_]·3H_2_O}_*n*_ stands out as a noteworthy coordination polymer with distinctive features. This study focuses on unraveling the mechanism underlying the combination of this coordination polymer with phosphors, specifically, YAG:Ce, NCS:Sm, and CAO:Mn. The intricate interplay between the {[Cu(μ_3_-dmg)(im)_2_]·3H_2_O}*n* coordination polymer, and these phosphors hold great promise for advancing our understanding of their synergistic behavior in luminescent applications. Herein when combined with the coordination polymer additive, the decay time kinetics of the three phosphors embedded in the PMMA thin film matrix were studied.

Potential donor–acceptor pairings and their decay patterns are shown in Table 5 for various composites made by gradually increasing the acceptor content while maintaining a constant donor quantity. The decay time data were given in terms of the donor’s excitation wavelength, which is 362 nm (see Figure S1). When the donor and acceptor amounts were equal, the average decay time values for YAG:Ce, NCS:Sm, and CAO:Mn along with PKY159 were found to be 9.62, 3.66, and 10.31 μs, respectively, and on the other hand, the average decay time decreased when we doubled the acceptor amount while maintaining a constant donor amount.

**Table 5:** Decay Times of the YAG:Ce, NCS:Sm, and CAO:Mn along with PKY159 Recorded on a Microsecond Scale in the Thin Film of PMMA

Composite	Donor	Acceptor	λ_max_^ex^ (nm)	λ_max_^em^ (nm)	τ_0_	Decay Time (μs)	Std. Dev. (μs)	Rel. (%)
YAG:Ce + PKY159	PKY159 (5 mg)	YAG:Ce (5 mg)	362	424	τ_1_	1.58	0.08	10.01
					τ_2_	10.51	0.03	89.99
					τ_avr_	**9.62**		
YAG:Ce + PKY159	PKY159 (5 mg)	YAG:Ce (10 mg)	362	424	τ_1_	3.15	0.07	29.44
					τ_2_	11.00	0.06	70.56
					τ_avr_	**8.69**		
NCS:Sm + PKY159	NCS:Sm (5 mg)	PKY159 (5 mg)	362	424	τ_1_	0.82	0.01	64.28
					τ_2_	8.77	0.07	35.72
					τ_avr_	**3.66**		
NCS:Sm + PKY159	NCS:Sm (5 mg)	PKY159 (10 mg)	362	424	τ_1_	0.80	0.01	65.31
					τ_2_	8.88	0.08	34.69
					τ_avr_	**3.61**		
CAO:Mn + PKY159	PKY159 (5 mg)	CAO:Mn (5 mg)	362	424	τ_1_	1.15	0.01	4.26
					τ_2_	10.72	0.08	95.74
					τ_avr_	**10.31**		
CAO:Mn + PKY159	PKY159 (5 mg)	CAO:Mn (10 mg)	362	424	τ_1_	1.30	0.01	35.47
					τ_2_	9.32	0.03	64.53
					τ_avr_	**6.47**		

We concentrated on the donor–acceptor approach, treating the MOF material as the donor and the phosphors as the acceptor, in order to determine the causes of the increase in the emission intensity of the phosphors embedded alongside the MOFs. The hypothesis states that energy transfer presents itself as an increase in acceptor emission, together with a decrease in fluorescence decay time and quenching of donor fluorescence if both parties are emitting particles.

An alternative explanation could be that the higher phosphor molecule concentration improves the effectiveness of energy transfer from the coordination polymer to the phosphor. This may cause the coordination polymer’s excited state to deplete more quickly, reducing the decay period. Exciton migration, or the movement of electron–hole pairs between phosphor molecules, may be more effective at higher phosphor concentrations. This migratory mechanism may cause the excited state to decay more quickly and the overall decay time to decrease. Dipole–dipole interactions between nearby molecules may become more intense due to the increased concentration of phosphor molecules. Shorter decay durations may arise from these interactions’ effects on the radiative and nonradiative decay processes. When the amount of phosphor is doubled while the coordination polymer remains constant, the emission intensity increases, and the decay time decreases, indicating complicated interactions between the two materials that may involve molecular contacts, energy transfer processes, and exciton dynamics.^7^

## 4 Conclusions

We investigated here the interesting combination of YAG:Ce, NCS:Sm, and CAO:Mn phosphors with the {[Cu(μ_3_-dmg)(im)_2_]·3H_2_O}_*n*_ coordination polymer. In particular, when it comes to luminous materials, comprehending the molecular features of this combination is essential to maximizing the synergistic effects of the coordinating polymers and phosphors. The unique optical and electronic characteristics of PKY159, along with the luminous qualities of YAG:Ce, NCS:Sm, and CAO:Mn, offer promising prospects for the creation of new materials with improved optical properties. To successfully integrate the PKY159 coordination polymer with these phosphors, we wanted to understand the coordination dynamics and electrical interactions that underlie this investigation. YAG:Ce, NCS:Sm, and CAO:Mn phosphors incorporated with PKY159 in the PMMA matrix exhibited an approximately 29-, 5-, and 27-fold increase in brightness, respectively, compared to their nonadditive forms. Under 460, 402, and 466 nm excitation in the range of 303–503 K, respectively, the luminescence thermal stability of PKY159-doped versions of all produced phosphors was investigated in the study. Higher thermal stability was demonstrated by an increase in the activation energy values seen in the data. It is evident from the data that blends of all phosphors, including coordination polymer, exhibit excellent color purity and good CIE chromaticity coordinates. The measured IQE of the doped YAG:Ce, NCS:Sm, and CAO:Mn mixes (95.6%, 66.3%, and 84.6%) is significantly higher than that of the additive-free form of the phosphors, and this increase is paralleled with the rise in steady-state measurements. When encapsulated in the PMMA in the proper ratio, the coordination polymer functions as a donor and phosphor as an acceptor, according to experimental investigations conducted on steady-state and decay time. When the quantity of acceptor was doubled, the microsecond time scale excited state lifetimes of NCS:Sm, YAG:Ce, and CAO:Mn decreased from 3.66 to 3.61, 9.62 to 8.69, and 10.31 to 6.47, respectively. The results of this study encourage the development of new materials with customized luminous properties for use in lighting, display, and other fields. Furthermore, the use of coordination polymers in this field is promising.

## Data Availability

Authors confirm that all relevant data are included in the article and/or its Supporting Information.

## Supporting Information Available

Figure S1. Decay curves of (a) YAG:Ce, (b) NCS:Sm, and (c) CAO:Mn along with PKY159 recorded in a microsecond scale in the thin film of PMMA upon excitation at 362 nm (PDF)

## References

[ref1] Murthy K. V. R. Phosphor for Optoelectronic Devices: A Review. J. Innov. Electron. Commun. Eng. 2013, 3 (1), 21–28.

[ref2] Ye S.; Xiao F.; Pan Y. X.; Ma Y. Y.; Zhang Q. Y. Phosphors in Phosphor-Converted White Light-Emitting Diodes: Recent Advances in Materials, Techniques and Properties. Mater. Sci. Eng. R Reports 2010, 71 (1), 1–34. 10.1016/j.mser.2010.07.001.

[ref3] Nair G. B.; Swart H. C.; Dhoble S. J. A Review on the Advancements in Phosphor-Converted Light Emitting Diodes (Pc-LEDs): Phosphor Synthesis, Device Fabrication and Characterization. Prog. Mater. Sci. 2020, 109, 100622 10.1016/j.pmatsci.2019.100622.

[ref4] Jin Y.; Hu Y.; Wu H.; Duan H.; Chen L.; Fu Y.; Ju G.; Mu Z.; He M. A Deep Red Phosphor Li2MgTiO4: Mn4+ Exhibiting Abnormal Emission: Potential Application as Color Converter for Warm w-LEDs. Chem. Eng. J. 2016, 288, 596–607. 10.1016/j.cej.2015.12.027.

[ref5] George N. C.; Denault K. A.; Seshadri R. Phosphors for Solid-State White Lighting. Annu. Rev. Mater. Res. 2013, 43, 481–501. 10.1146/annurev-matsci-073012-125702.

[ref6] Gupta I.; Singh S.; Bhagwan S.; Singh D. Rare Earth (RE) Doped Phosphors and Their Emerging Applications: A Review. Ceram. Int. 2021, 47 (14), 19282–19303. 10.1016/j.ceramint.2021.03.308.

[ref7] Yildirim B.; Dalmis R.; Ertekin K.; Birlik I.; Azem F. A. Enhancing Optical Properties of Lu_3_Al_5_O_12_:Ce^3+^ by Cost-Effective Silica-Based Photonic Crystals. J. Mater. Sci. Mater. Electron. 2020, 31 (13), 10267–10278. 10.1007/s10854-020-03573-7.

[ref8] Zeyrek Ongun M. Investigation of Brightness and Decay Characteristics of YAG:Ce^3+^, Ca-α-Sialon: Eu^2+^ and CaAl_12_O_19_:Mn^4+^ Phosphors Incorporated with Ni-Doped SnO_2_ Particles. J. Lumin. 2021, 240, 118405 10.1016/j.jlumin.2021.118405.

[ref9] Clabau F.; Rocquefelte X.; Jobic S.; Deniard P.; Whangbo M.-H.; Garcia A.; Le Mercier T. Mechanism of Phosphorescence Appropriate for the Long-Lasting Phosphors Eu^2+^-Doped SrAl_2_O_4_ with Codopants Dy^3+^ and B^3+^. Chem. Mater. 2005, 17 (15), 3904–3912. 10.1021/cm050763r.

[ref10] Yang W.-J.; Luo L.; Chen T.-M.; Wang N.-S. Luminescence and Energy Transfer of Eu-and Mn-Coactivated CaAl_2_Si_2_O_8_ as a Potential Phosphor for White-Light UVLED. Chem. Mater. 2005, 17 (15), 3883–3888. 10.1021/cm050638f.

[ref11] Park J. H.; Kwon M. S. Sol-Gel Synthesis of CaAl_12_O_19_:Mn Phosphor and Red Luminescence by near-Ultraviolet/Blue Excitation. Electron. Mater. Lett. 2010, 6, 13–15. 10.3365/eml.2010.03.013.

[ref12] Ueda J.; Tanabe S. Review of Luminescent Properties of Ce^3+^-Doped Garnet Phosphors: New Insight into the Effect of Crystal and Electronic Structure. Opt. Mater. X 2019, 1, 100018 10.1016/j.omx.2019.100018.

[ref13] Kaur H.; Jayasimhadri M.; Sahu M. K.; Rao P. K.; Reddy N. S. Synthesis of Orange Emitting Sm^3+^ Doped Sodium Calcium Silicate Phosphor by Sol-Gel Method for Photonic Device Applications. Ceram. Int. 2020, 46 (16), 26434–26439. 10.1016/j.ceramint.2020.04.224.

[ref14] Inkrataite G.; Zabiliute-Karaliune A.; Aglinskaite J.; Vitta P.; Kristinaityte K.; Marsalka A.; Skaudzius R. Study of YAG:Ce and Polymer Composite Properties for Application in LED Devices. ChemPlusChem. 2020, 85 (7), 1504–1510. 10.1002/cplu.202000318. 32644307

[ref15] Song J.; Jiang C.; Liu X.; Yang Z.; Guan R.; Wang M.; Chai S. PL Intensity of YAG:Ce^3+^ Phosphor Powder Enhanced by Ag NPs with Soluble and Cracked Polymer Spacer. Opt. Mater. (Amst). 2019, 95, 109233 10.1016/j.optmat.2019.109233.

[ref16] Wu H.; Lu T.; Wei N.; Lu Z.; Chen X.; Guan Y.; Zhao Y.; Qi J.; Shi Q.; Xie X.; Zhang W. Photoluminescence Enhancement of YAG:Ce Nanophosphors with SiO_2_ Additions. J. Mater. Sci. Mater. Electron. 2015, 26, 2451–2456. 10.1007/s10854-015-2705-0.

[ref17] Ueda J.; Miyano S.; Tanabe S. Formation of Deep Electron Traps by Yb^3+^ Codoping Leads to Super-Long Persistent Luminescence in Ce^3+^-Doped Yttrium Aluminum Gallium Garnet Phosphors. ACS Appl. Mater. Interfaces 2018, 10 (24), 20652–20660. 10.1021/acsami.8b02758. 29791129

[ref18] Mickens M. A.; Assefa Z. Tunable Luminescence and White Light Emission of Novel Multiphase Sodium Calcium Silicate Nanophosphors Doped with Ce^3+^, Tb^3+^, and Mn^2+^ Ions. J. Lumin. 2014, 145, 498–506. 10.1016/j.jlumin.2013.07.053.

[ref19] Wu Y.; Zhuang Y.; Xie R.-J.; Ruan K.; Ouyang X. Novel Mn 4+ Doped Red Phosphors Composed of MgAl_2_O_4_ and CaAl_12_O_19_ Phases for Light-Emitting Diodes. Dalt. Trans. 2020, 49 (11), 3606–3614. 10.1039/D0DT00118J. 32129394

[ref20] Brik M. G.; Pan Y. X.; Liu G. Spectroscopic and Crystal Field Analysis of Absorption and Photoluminescence Properties of Red Phosphor CaAl_12_O_19_:Mn^4+^ Modified by MgO. J. Alloys Compd. 2011, 509 (5), 1452–1456. 10.1016/j.jallcom.2010.11.117.

[ref21] Pan Y. X.; Liu G. K. Influence of Mg^2+^ on Luminescence Efficiency and Charge Compensating Mechanism in Phosphor CaAl_12_O_19_:Mn^4+^. J. Lumin. 2011, 131 (3), 465–468. 10.1016/j.jlumin.2010.11.014.

[ref22] Zhao Y.; Shi L.; Han Y.; Li H.; Ji Z.; Zhang Z. Luminescent Properties of Zn2+-Doped CaAl_12_O_19_:Mn^4+^ Deep-Red Phosphor for Indoor Plant Cultivation. Ceram. Int. 2019, 45 (7), 8265–8270. 10.1016/j.ceramint.2019.01.132.

[ref23] Robin A. Y.; Fromm K. M. Coordination Polymer Networks with O-and N-Donors: What They Are, Why and How They Are Made. Coord. Chem. Rev. 2006, 250 (15–16), 2127–2157. 10.1016/j.ccr.2006.02.013.

[ref24] Wang M.-S.; Guo S.-P.; Li Y.; Cai L.-Z.; Zou J.-P.; Xu G.; Zhou W.-W.; Zheng F.-K.; Guo G.-C. A Direct White-Light-Emitting Metal- Organic Framework with Tunable Yellow-to-White Photoluminescence by Variation of Excitation Light. J. Am. Chem. Soc. 2009, 131 (38), 13572–13573. 10.1021/ja903947b. 19772357

[ref25] Zhang X.; Wang W.; Hu Z.; Wang G.; Uvdal K. Coordination Polymers for Energy Transfer: Preparations, Properties, Sensing Applications, and Perspectives. Coord. Chem. Rev. 2015, 284, 206–235. 10.1016/j.ccr.2014.10.006.

[ref26] Tang Y.; Wu H.; Cao W.; Cui Y.; Qian G. Luminescent Metal-Organic Frameworks for White LEDs. Adv. Opt. Mater. 2021, 9 (23), 2001817 10.1002/adom.202001817.

[ref27] Lustig W. P.; Li J. Luminescent Metal-Organic Frameworks and Coordination Polymers as Alternative Phosphors for Energy Efficient Lighting Devices. Coord. Chem. Rev. 2018, 373, 116–147. 10.1016/j.ccr.2017.09.017.

[ref28] Zhou B.; Yan D. Long Persistent Luminescence from Metal-Organic Compounds: State of the Art. Adv. Funct. Mater. 2023, 33 (19), 2300735 10.1002/adfm.202300735.

[ref29] Ahmed I.; Jhung S. H. Composites of Metal-Organic Frameworks: Preparation and Application in Adsorption. Mater. today 2014, 17 (3), 136–146. 10.1016/j.mattod.2014.03.002.

[ref30] Li S.; Huo F. Metal-Organic Framework Composites: From Fundamentals to Applications. Nanoscale 2015, 7 (17), 7482–7501. 10.1039/C5NR00518C. 25871946

[ref31] Stock N.; Biswas S. Synthesis of Metal-Organic Frameworks (MOFs): Routes to Various MOF Topologies, Morphologies, and Composites. Chem. Rev. 2012, 112 (2), 933–969. 10.1021/cr200304e. 22098087

[ref32] Gao R.; Kodaimati M. S.; Yan D. Recent Advances in Persistent Luminescence Based on Molecular Hybrid Materials. Chem. Soc. Rev. 2021, 50 (9), 5564–5589. 10.1039/D0CS01463J. 33690765

[ref33] Chen W.; Zhuang Y.; Wang L.; Lv Y.; Liu J.; Zhou T.-L.; Xie R.-J. Color-Tunable and High-Efficiency Dye-Encapsulated Metal-Organic Framework Composites Used for Smart White-Light-Emitting Diodes. ACS Appl. Mater. Interfaces 2018, 10 (22), 18910–18917. 10.1021/acsami.8b04937. 29770686

[ref34] Chen M.; Sun R.; Ye Y.; Tang H.; Dong X.; Yan J.; Wang K.; Zhou Q.; Wang Z. Application of a Novel Red-Emitting Cationic Iridium (III) Coordination Polymer in Warm White Light-Emitting Diodes. Opt. Mater. (Amst). 2018, 76, 141–146. 10.1016/j.optmat.2017.12.005.

[ref35] Yildirim B.; Keskin O. Y.; Dalmis R.; Birlik I.; Azem F. A.; Ertekin K. Investigation of TiO_2_ and Ce^3+^-Activated TiO_2_ Particles on Optical Properties of the PMMA Embedded YAG:Ce^3+^ and LuAG:Ce^3+^. Opt. Mater. (Amst). 2022, 133, 112905 10.1016/j.optmat.2022.112905.

[ref36] Verma T.; Agrawal S. Photoluminescence Characteristics of Sm^3+^ and Eu^3+^ Doped Yttrium Oxide Phosphors. J. Mater. Sci. Mater. Electron. 2018, 29, 13397–13406. 10.1007/s10854-018-9465-6.

[ref37] Wang W.; Zhu P. Red Photoluminescent Eu^3+^-Doped Y_2_O_3_ Nanospheres for LED-Phosphor Applications: Synthesis and Characterization. Opt. Express 2018, 26 (26), 34820–34829. 10.1364/OE.26.034820. 30650899

[ref38] Zhu P.; Wang W.; Zhu H.; Vargas P.; Bont A. Optical Properties of Eu^3+^-Doped Y_2_O_3_ Nanotubes and Nanosheets Synthesized by Hydrothermal Method. IEEE Photonics J. 2018, 10 (1), 1–10. 10.1109/JPHOT.2018.2797950.

[ref39] Shi Y.; Yang Z.; Wang W.; Zhu G.; Wang Y. Novel Red Phosphors Na_2_CaSiO_4_:Eu^3+^ for Light-Emitting Diodes. Mater. Res. Bull. 2011, 46 (7), 1148–1150. 10.1016/j.materresbull.2011.03.001.

[ref40] Zhang W.; He M.; Jia J.; Liu C.; Xu Q.; Qiao X.; Fan X. Monodisperse CaAl_12_O_19_:Mn^4+^ Microspheres for Full-Spectrum White LEDs. J. Sol-Gel Sci. Technol. 2022, 104 (2), 434–446. 10.1007/s10971-022-05954-2.

[ref41] Sun C.-Y.; Wang X.-L.; Zhang X.; Qin C.; Li P.; Su Z.-M.; Zhu D.-X.; Shan G.-G.; Shao K.-Z.; Wu H.; Li J. Efficient and Tunable White-Light Emission of Metal-Organic Frameworks by Iridium-Complex Encapsulation. Nat. Commun. 2013, 4 (1), 2717 10.1038/ncomms3717. 24212250 PMC3831296

[ref42] Xia Y.-P.; Wang C.-X.; An L.-C.; Zhang D.-S.; Hu T.-L.; Xu J.; Chang Z.; Bu X.-H. Utilizing an Effective Framework to Dye Energy Transfer in a Carbazole-Based Metal-Organic Framework for High Performance White Light Emission Tuning. Inorg. Chem. Front. 2018, 5 (11), 2868–2874. 10.1039/C8QI00747K.

[ref43] Yaman P. K.; Demir S.; Denizaltı S.; Erer H.; Yesilel O. Z. A Copper (Ii) Metal-Organic Framework with 2, 2-Dimethylglutarate and Imidazole Ligands: Synthesis, Characterization and Catalytic Performance for Cycloaddition of CO_2_ to Epoxides. New J. Chem. 2024, 48 (4), 1850–1855. 10.1039/D3NJ04256A.

[ref44] Sakar N.; Gergeroglu H.; Akalin S. A.; Oguzlar S.; Yildirim S. Synthesis, Structural and Optical Characterization of Nd:YAG Powders via Flame Spray Pyrolysis. Opt. Mater. (Amst). 2020, 103, 109819 10.1016/j.optmat.2020.109819.

[ref45] Oguzlar S.; Ongun M. Z.; Deliormanlı A. M. Improvement of Optical Properties of One-Dimensional CaAl_12_O_19_:Mn^4+^ Phosphor via Er^3+^-and Tb^3+^-Doped Bioactive Glass Powders. Opt. Mater. (Amst). 2021, 117, 111201 10.1016/j.optmat.2021.111201.

[ref46] Yucai H. U.; Yimin L. Ü.; Xuehua Y. U.; Li Z.; Junsheng Y. U. Synthesis and Characterization of YAG:Ce^3+^ Fluorescence Powders by Co-Precipitation Method. J. Rare Earths 2010, 28, 303–307. 10.1016/S1002-0721(10)60283-9.

[ref47] Lee H.-M.; Cheng C.-C.; Huang C.-Y. The Synthesis and Optical Property of Solid-State-Prepared YAG:Ce Phosphor by a Spray-Drying Method. Mater. Res. Bull. 2009, 44 (5), 1081–1085. 10.1016/j.materresbull.2008.10.006.

[ref48] Masenelli B.; Mollet O.; Boisron O.; Canut B.; Ledoux G.; Bluet J.-M.; Mélinon P.; Dujardin C.; Huant S. YAG: Ce Nanoparticle Lightsources. Nanotechnology 2013, 24 (16), 165703 10.1088/0957-4484/24/16/165703. 23535555

[ref49] Wang L.; Zhao F.; Yang X.; Pan C.; Huang H. Property of YAG:Ce^3+^ Nanophosphors Prepared by Solvothermal Method Using Triethylene-Tetramine as a Reaction Solvent. RSC Adv. 2015, 5 (33), 26339–26345. 10.1039/C5RA00580A.

[ref50] Zhao Y.; Xu H.; Zhang X.; Zhu G.; Yan D.; Yu A. Facile Synthesis of YAG:Ce^3+^ Thick Films for Phosphor Converted White Light Emitting Diodes. J. Eur. Ceram. Soc. 2015, 35 (13), 3761–3764. 10.1016/j.jeurceramsoc.2015.05.017.

[ref51] Yun Y.-H.; Yoon C.-H.; Oh J.-S.; Kim S.-B.; Kang B.-A.; Hwang K.-S. Waste Fluorescent Glass and Shell Derived Glass-Ceramics. J. Mater. Sci. 2002, 37, 3211–3215. 10.1023/A:1016118613911.

[ref52] Fu M.; Wu Q.; Gu C.; Hu S.; Lu S.; Wang B.; Hong Y.; Wang Y. Energy Transfer in Sm^3+^/Eu^3+^ Doped Li_6_Gd(BO_3_)_3_ Orange-Red Phosphor. Vacuum 2023, 214, 112147 10.1016/j.vacuum.2023.112147.

[ref53] Oguzlar S.; Zeyrek Ongun M.; Dogan Tunc I.; Erol M. Development of High Luminous Efficacy Red-Emitting CaAl_12_O_19_:Mn^4+^ Phosphor Using Al-and K-Doped ZnO NWs/CFs. J. Mater. Sci. Mater. Electron. 2023, 34 (16), 1267 10.1007/s10854-023-10685-3.

[ref54] Bispo-Jr A. G.; Saraiva L. F.; Lima S. A. M.; Pires A. M.; Davolos M. R. Recent Prospects on Phosphor-Converted LEDs for Lighting, Displays, Phototherapy, and Indoor Farming. J. Lumin. 2021, 237, 118167 10.1016/j.jlumin.2021.118167.

[ref55] Di X.; He X.; Jiang J.; Li P.; Xiang W.; Liang X.; Shen T. Facile Fabrication of Eu^3+^ Activated YAG:Ce^3+^ Glass Ceramics Exhibiting High Thermal Stability and Tunable Luminescence for Warm White LEDs. J. Mater. Sci. Mater. Electron. 2017, 28, 8611–8620. 10.1007/s10854-017-6585-3.

[ref56] Lu T.; Ma Z.; Du C.; Fang Y.; Wu H.; Jiang Y.; Wang L.; Dai L.; Jia H.; Liu W.; Chen H. Temperature-Dependent Photoluminescence in Light-Emitting Diodes. Sci. Rep. 2014, 4 (1), 6131 10.1038/srep06131. 25139682 PMC5381404

[ref57] Lin Y.-C.; Bettinelli M.; Karlsson M. Unraveling the Mechanisms of Thermal Quenching of Luminescence in Ce^3+^-Doped Garnet Phosphors. Chem. Mater. 2019, 31 (11), 3851–3862. 10.1021/acs.chemmater.8b05300.

[ref58] Kong L.; Liu Y.; Dong L.; Zhang L.; Qiao L.; Wang W.; You H. Enhanced Red Luminescence in CaAl_12_O_19_:Mn^4+^ via Doping Ga^3+^ for Plant Growth Lighting. Dalt. Trans. 2020, 49 (6), 1947–1954. 10.1039/C9DT04086B. 31976498

[ref59] Liu Y.; Zou J.; Shi M.; Yang B.; Han Y.; Li W.; Wang Z.; Zhou H.; Li M.; Jiang N. Effect of Gallium Ion Content on Thermal Stability and Reliability of YAG:Ce Phosphor Films for White LEDs. Ceram. Int. 2018, 44 (1), 1091–1098. 10.1016/j.ceramint.2017.10.056.

[ref60] Huang X.; Liang J.; Rtimi S.; Devakumar B.; Zhang Z. Ultra-High Color Rendering Warm-White Light-Emitting Diodes Based on an Efficient Green-Emitting Garnet Phosphor for Solid-State Lighting. Chem. Eng. J. 2021, 405, 126950 10.1016/j.cej.2020.126950.

[ref61] Liang J.; Devakumar B.; Sun L.; Wang S.; Sun Q.; Huang X. Full-Visible-Spectrum Lighting Enabled by an Excellent Cyan-Emitting Garnet Phosphor. J. Mater. Chem. C 2020, 8 (14), 4934–4943. 10.1039/D0TC00006J.

[ref62] Ogi T.; Nandiyanto A. B. D.; Wang W.-N.; Iskandar F.; Okuyama K. Direct Synthesis of Spherical YAG:Ce Phosphor from Precursor Solution Containing Polymer and Urea. Chem. Eng. J. 2012, 210, 461–466. 10.1016/j.cej.2012.09.033.

