# Supplementary material for: Improved Optical Performance Analysis of YAG:Ce, NCS:Sm, and CAO:Mn Phosphors Physically Integrated with Metal–Organic Frameworks
Source: ACS Omega. 2024 Oct 9;9(42):43219–32. doi: 10.1021/acsomega.4c07786 (PMC11500154; doi:10.1021/acsomega.4c07786)
Supplement: Supplementary file 1 — ao4c07786_si_001.pdf [file ao4c07786_si_001.pdf]

Supporting Information

Improved Optical Performance Analysis of YAG:Ce, NCS:Sm, CAO:Mn Phosphors Physically Integrated with Metal-Organic Frameworks

Sibel Oguzlar[a*], Merve Zeyrek Ongun[b], Pelin Köse Yaman[c], Mustafa Erol[d]

[a*]Dokuz Eylul University, Center for Fabrication and Application of Electronic Materials, 35390, Izmir, Turkey

[b]Dokuz Eylul University, Izmir Vocational High School, Chemistry and Chemical Processing Technologies Department, Chemical Technology Program, 35380, Izmir, Turkey

[c]Dokuz Eylul University, Department of Chemistry, Faculty of Science, 35390, Izmir, Turkey

[d]Dokuz Eylul University, Department of Metallurgical and Materials Engineering, 35390, Izmir, Turkey

List of Figures

Figure S1. Decay curves of **(a)** YAG:Ce, **(b)** NCS:Sm, and **(c)** CAO:Mn along with PKY159 recorded in a microsecond scale in the thin film of PMMA upon excitation at 362 nm.

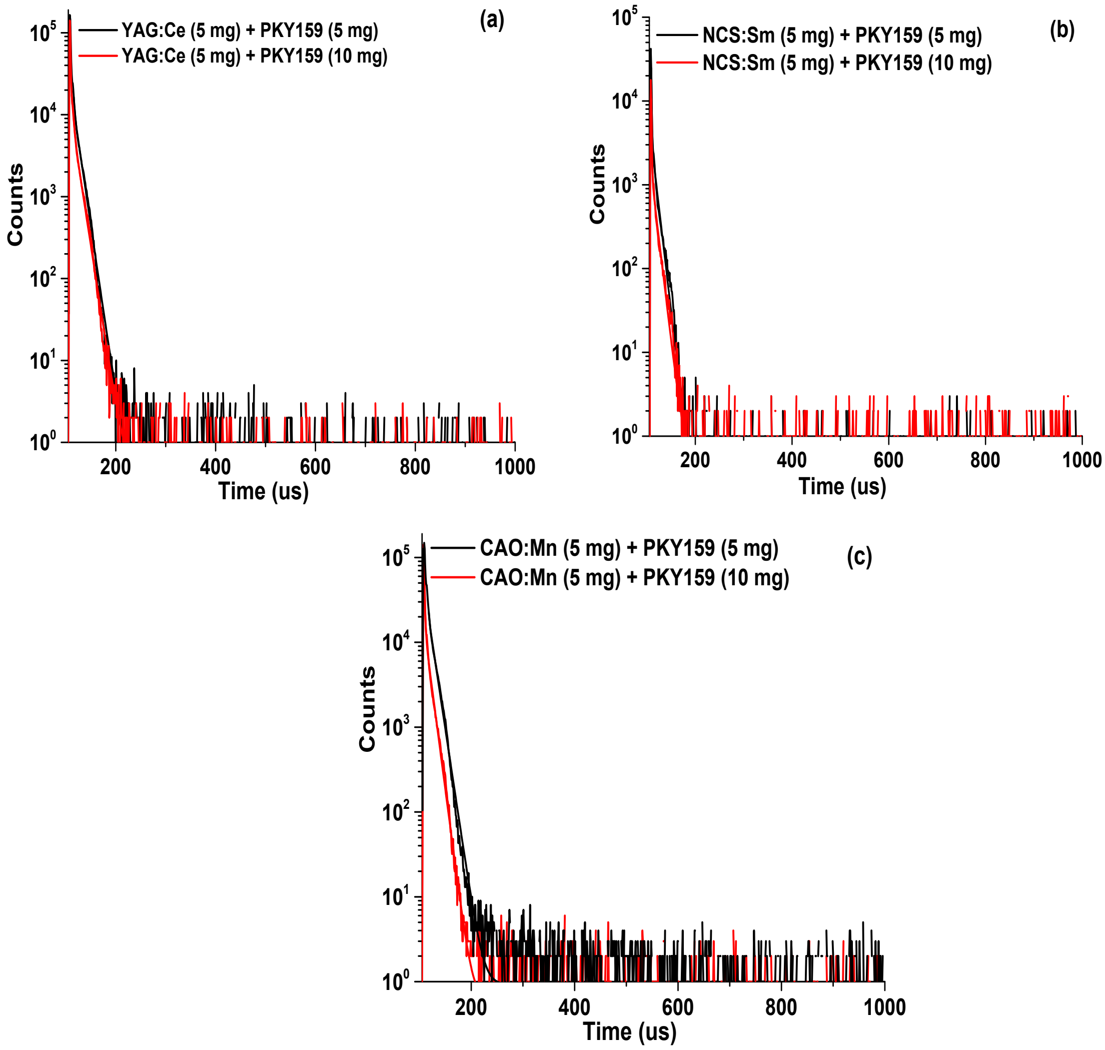

Figure S1. Decay curves of **(a)** YAG:Ce, **(b)** NCS:Sm, and **(c)** CAO:Mn along with PKY159 recorded in a microsecond scale in the thin film of PMMA upon excitation at 362 nm.
